# Genomic context analysis in Archaea suggests previously unrecognized links between DNA replication and translation

**DOI:** 10.1186/gb-2008-9-4-r71

**Published:** 2008-04-09

**Authors:** Jonathan Berthon, Diego Cortez, Patrick Forterre

**Affiliations:** 1 Univ. Paris-Sud 11, CNRS, UMR8621, Institut de Génétique et Microbiologie, 91405 Orsay CEDEX, France; 2 Laboratory of Protein Chemistry and Engineering, Department of Genetic Resources Technology, Faculty of Agriculture, Kyushu University, 6-10-1 Hakozaki, Higashi-ku, Fukuoka-shi, Fukuoka 812-8581, Japan; 3 Institut Pasteur, rue Dr. Roux, 75724 Paris CEDEX 15, France

## Abstract

**Background:**

Comparative analysis of genomes is valuable to explore evolution of genomes, deduce gene functions, or predict functional linking between proteins. Here, we have systematically analyzed the genomic environment of all known DNA replication genes in 27 archaeal genomes to infer new connections for DNA replication proteins from conserved genomic associations.

**Results:**

Two distinct sets of DNA replication genes frequently co-localize in archaeal genomes: the first includes the genes for PCNA, the small subunit of the DNA primase (PriS), and Gins15; the second comprises the genes for MCM and Gins23. Other genomic associations of genes encoding proteins involved in informational processes that may be functionally relevant at the cellular level have also been noted; in particular, the association between the genes for PCNA, transcription factor S, and NudF. Surprisingly, a conserved cluster of genes coding for proteins involved in translation or ribosome biogenesis (S27E, L44E, aIF-2 alpha, Nop10) is almost systematically contiguous to the group of genes coding for PCNA, PriS, and Gins15. The functional relevance of this cluster encoding proteins conserved in Archaea and Eukarya is strongly supported by statistical analysis. Interestingly, the gene encoding the S27E protein, also known as metallopanstimulin 1 (MPS-1) in human, is overexpressed in multiple cancer cell lines.

**Conclusion:**

Our genome context analysis suggests specific functional interactions for proteins involved in DNA replication between each other or with proteins involved in DNA repair or transcription. Furthermore, it suggests a previously unrecognized regulatory network coupling DNA replication and translation in Archaea that may also exist in Eukarya.

## Abstract 2

Specific functional interactions of proteins involved in DNA replication and/or DNA repair or transcription might occur in Archaea, suggesting a previously unrecognized regulatory network coupling DNA replication and translation, which might also exist in Eukarya.

## Background

Alignment of prokaryotic genomes revealed that synteny is globally weak, indicating that bacterial and archaeal chromosomes experience continuous remodeling [1-3]. A few operons encoding physically interacting proteins involved in fundamental processes have been preserved between Archaea and Bacteria in the course of evolution (for example, operons encoding ribosomal proteins, RNA polymerase subunits, or ATP synthase subunits) [1-3]. Most gene strings are only conserved in closely related genomes or exhibit a patchy distribution among genomes in one large group of organisms (for example, in Archaea). Therefore, gene associations that are conserved between distantly related organisms should confer some selective advantage. The co-localization of a particular group of genes may optimize their co-regulation at the transcriptional level [4,5] or facilitate the assembly of their products in large protein complexes [6]. A corollary of this statement is that characterization of evolutionarily conserved gene clusters can be used to infer functional linkage of proteins (that is, physical interaction or participation in a common structural complex, metabolic pathway, or biological process). Various comparative genomics methods that exploit gene context are commonly used. These approaches analyze protein and domain fusion or gene neighborhood (groups of genes found in putative operons or divergently transcribed gene pairs) to predict functions for, and interactions between, the encoded proteins (reviewed in [2,7-10]). A dramatic example of a discovery based on genome context analysis is the identification in Archaea and Bacteria of proteins associated with the specific DNA repeats known as CRISPR [11]. These *cas *proteins (for CRISPR associated proteins), which were first proposed to be members of a putative DNA repair system [12], are probable actors in a nucleic-acid based 'immunity' system [13]. Comparative analysis of genomes has been especially helpful in Archaea for functional prediction of uncharacterized proteins in the absence of genetic studies (reviewed in [14,15]). For instance, this strategy has allowed the computational prediction and subsequent experimental confirmation of the archaeal exosome [16,17] and of novel proteins associated with the Mre11/Rad50 complex [18,19].

Many putative DNA replication proteins have been identified in archaeal genomes by similarities with their eukaryotic counterparts known experimentally to be involved in DNA replication (for a review, see [20]). Most of these proteins have now been purified from one or more Archaea and characterized to various extents *in vitro *(reviewed in [20]). Several examples of physical and/or functional interactions between archaeal DNA replication proteins have now emerged from biochemical studies (reviewed in [20]), supporting the idea that these proteins are indeed working together at the replication fork. A few clusters of genes encoding DNA replication proteins have been previously reported in *Pyrococcus *and *Sulfolobus *genomes [21-24]; in one case, the gene association correlates with protein physical interaction [24]. This suggests that systematic identification of clusters of genes encoding DNA replication proteins in the expanding collection of archaeal genomes could identify gene associations connecting genome organization to functional interactions of proteins that could be relevant *in vivo*. More importantly, comparative genomic analyses could be used to determine the most significant interactions, that is, those that appear to be recurrent in the genomes of evolutionarily diverse Archaea.

Here, we have performed a systematic genome context analysis of genes encoding DNA replication proteins in 27 completely sequenced archaeal genomes. Our results show that a subset of genes encoding DNA replication proteins often co-localize, that is, these genes are arranged in operon-like structures (contiguous or adjacent genes in the same transcriptional orientation) that are preserved between distant lineages (as for the majority of the cases discussed here), or they lie in a common chromosomal region less than 5 kilobases away from each other. Some of these associations are conserved between distant lineages, indicating that they reflect a functional and possibly a physical interaction between the gene products. In particular, we identified two conserved genomic associations of DNA replication genes that suggest a functional connection between the PCNA, the DNA primase and the MCM helicase via the GINS complex. We also observed that the gene for PCNA is linked to the gene coding for the transcription factor S (TFS) in 12 out of the 27 analyzed genomes, as well as to a gene encoding the ADP-ribose pyrophosphatase NudF in 8 genomes, pointing toward the existence of cross-talk between DNA replication, DNA repair, and transcription. In addition, we noticed that the gene encoding the initiator protein Cdc6 is usually adjacent to a predicted origin of replication, sometimes together with or close to the gene coding for the small subunit of DNA polymerase (Pol)D (DP1) in euryarchaeal genomes, suggesting that PolD may be recruited by Cdc6 at the origin of replication. Moreover, some proteins without clear functional assignments (an oligonucleotide/oligosaccharide-binding (OB)-fold containing protein, a recently described new GTPase, DnaG) are encoded by genes that co-localize with DNA replication genes, suggesting that they may be involved in DNA transaction processes. Surprisingly, our analysis also reveals a widely conserved clustering of a particular set of genes coding for DNA replication proteins (Gins15, PCNA and/or the DNA primase small subunit (PriS)) with a special set of genes encoding proteins related to the ribosome (L44E, S27E, aIF-2 alpha, Nop10). This cluster is strongly supported by a statistical analysis based on the actual distribution of gene clusters in the set of genomes analyzed in this study, suggesting the existence of a previously unrecognized regulatory network coupling DNA replication and translation in Archaea.

## Results and discussion

### Systematic identification of DNA replication genes in archaeal genomes

**Figure 1:**
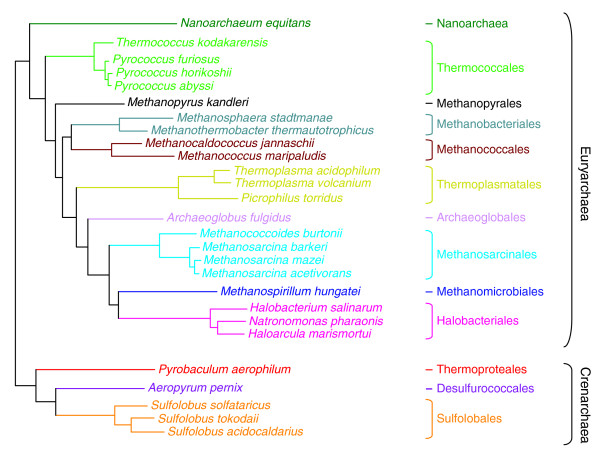
Phylogeny of the Archaea whose genomes have been analyzed in this study. This unrooted tree (kindly provided by Céline Brochier) is based on the concatenation of archaeal ribosomal proteins (see [73] for details). The parasitic archaeon *N. equitans *is placed with Euryarchaeota in accordance with the hypothesis that it likely represents a fast-evolving euryarchaeal lineage [34].

We have performed an exhaustive search of all known putative DNA replication genes in the 27 archaeal genomes available at the NCBI [25] as of 10 April 2006. These genomes include 5 genomes of Crenarchaea and 22 genomes of Euryarchaea, and are distributed among 13 different archaeal orders (Figure 1). Our list of DNA replication genes includes all genes coding for archaeal proteins or subunits of complexes corresponding to eukaryotic homologs known to be involved in DNA replication: the initiation factor Cdc6 (Orc1); PolB; the helicase MCM; the sliding clamp PCNA; the clamp-loader replication factor C (RFC); the DNA primase; the single-stranded binding protein RPA (or SSB in Crenarchaea); the DNA ligase; the RNase HII; the flap endonuclease FEN-1; and the two Gins subunits (Gins15 and Gins23). We have added to this list PolD (absent from hyperthermophilic Crenarchaea), since its genes are located close to the replication origin in Thermococcales [22] and because this enzyme is essential for *Halobacterium *sp. NRC-1 survival according to recent genetic data [26]. We have also included in our list the DNA topoisomerase VI (Topo VI) since this enzyme is the only DNA topoisomerase known in Archaea that can relax positive superturns, an essential function for DNA replication [27]. First, the 27 archaeal genomes available at the NCBI were searched to retrieve the entries of all the annotated DNA replication proteins (see Materials and methods) encoded by these genomes. Then, systematic BLASTP searches were carried out with several seeds for each protein in order to verify the annotations and to look for missing proteins (see Materials and methods); Additional data file 1 provides a table listing all putative DNA replication proteins identified and used in our analysis.

DNA replication proteins are encoded by a set of genes that is present in all archaeal genomes (sometimes with several paralogues), with the exception of PolD, which is absent in hyperthermophilic Crenarchaea; Gins23, which has only been detected in Crenarchaea and Thermococcales; RPA, which is absent in hyperthermophilic Crenarchaea; and the crenarchaeal SSB, which is currently restricted to Crenarchaea and Thermoplasmatales. We noticed a few interesting instances of missing DNA replication genes. In particular, we and others failed to detect a RPA or a SSB homolog in *Pyrobaculum aerophilum *[28,29] and this study) and a Cdc6/Orc1 homolog in *Methanopyrus kandleri *([30,31] and this study). On the other hand, we retrieved a Cdc6-like homolog that is related to the putative origin initiator protein of *Methanocaldococcus jannaschii *[32] in the genome of *Methanococcus maripaludis*. Moreover, we detected only one primase gene in *Nanoarchaeum equitans*; alignment of the amino acid sequence of *N. equitans *primase with other members of the archaeo-eukaryotic primase superfamily shows that it corresponds to the fusion of the amino-terminal region of the small subunit with the carboxy-terminal region of the large subunit [33]. Thus, the primase of *N. equitans *could be an interesting model to study the mechanism of action of this protein *in vitro*. Finally, the genome of *Methanococcoides burtonii *does not harbor any identifiable gene encoding the small non-catalytic subunit of PolD (DP1), whilst the gene encoding the large catalytic subunit (DP2) is present. It would be of particular interest to get insight into the functional properties of the *M. burtonii *PolD to unravel whether or not a core version of PolD exhibits the expected features, given that the interaction between the two subunits has been shown to be essential for full enzymatic activities of the canonical form [21].

### Genes encoding subunits of heteromultimeric DNA replication proteins rarely associate

Several DNA replication factors are formed by the association of two or more different protein subunits (that is, these DNA replication factors are heteromultimeric proteins), including RFC (RFC-s and RFC-l), primase (PriS and PriL), the PolD holoenzyme (DP1 and DP2), and Topo VI (A and B subunits). We did not detect any obvious trend of association for the genes encoding different subunits of heteromultimeric proteins among archaeal genomes, except for the genes encoding the Topo VI subunits and the genes for the RFC subunits. The genes encoding the two subunits of Topo VI are contiguous in all Archaea, except for *N. equitans*, Methanococcales, *Archaeoglobus fulgidus *and *Methanopyrus kandleri*, whereas the genes encoding the large and small subunits of RFC co-localize in Crenarchaea, Thermococcales, Methanobacteriales and *M. kandleri *(see Additional data file 2 for illustrations). Interestingly, the genes encoding the two subunits of Topo VI are contiguous to the genes encoding the two subunits of DNA gyrase (of bacterial origin) in all halophilic Archaea and in Methanosarcinales, suggesting a co-regulation of the two type II DNA topoisomerases that was selected after the transfer of the bacterial enzyme into its archaeal host. The genes encoding the two subunits of PolD are adjacent in Thermococcales only, and those for the two subunits of DNA primase co-localize in Thermococcales and Methanobacteriales; the primase genes are fused in *N. equitans *as previously mentioned (Additional data file 2). The genes encoding the three subunits of the heterotrimeric RPA found in Thermococcales (RPA41, RPA32, and RPA14) are clustered in the four completely sequenced genomes presently known, whereas the genes encoding RPA homologs present in other euryarchaeal genomes never associate. Finally, the genes encoding the two Gins proteins in Crenarchaea and Thermococcales are never adjacent. The tendency for genes encoding different subunits of DNA replication factors to co-localize is, therefore, very different from one gene to the other, a first indication that the observed gene associations are not random.

In the course of this work, we noticed that co-localization of DNA replication genes - encoding different subunits of heteromultimeric proteins (see above) or encoding different proteins (see below) - are more frequent in some genomes than in others. They are especially rare in *N. equitans *since all the gene strings that are conserved in all other archaeal genomes are disrupted in this archaeon. It is likely that these disruptions are due to extensive genome rearrangements that occurred in this species because *N. equitans *is a parasitic organism that has adapted to its lifestyle by extensive genome reduction, including the split of several genes [15,34]. At the other end of the spectrum, we observed that the clustering of DNA replication genes occurs very frequently in Thermococcales. Indeed, all genes encoding different subunits of heteromultimeric DNA replication proteins are contiguous in this lineage, except those encoding the two subunits of the archaeal GINS complex.

### Conserved gene clusters suggest functional linkage between PCNA, DNA primase, GINS, and MCM

**Figure 2:**
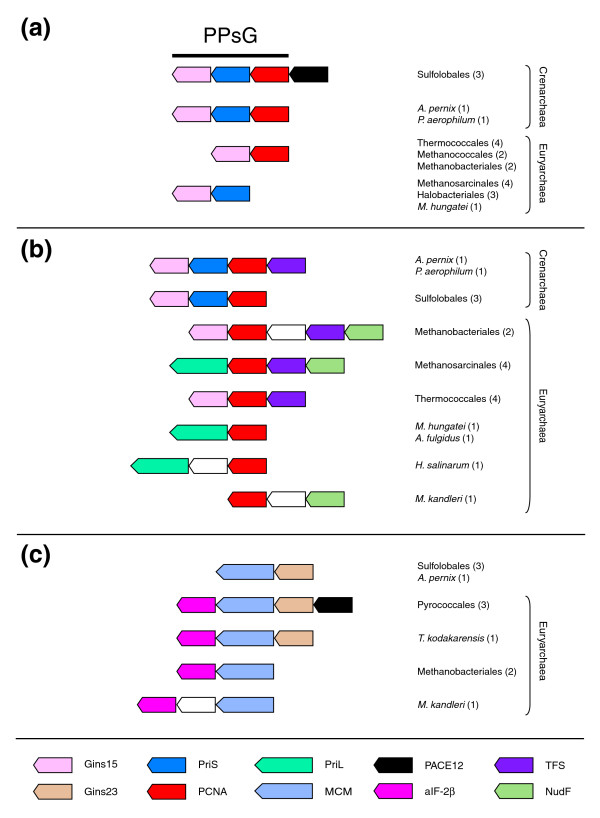
Conserved genomic context of three DNA replication genes in archaeal genomes. This figure highlights the genome context of three DNA replication genes that recurrently associate with a particular set of genes in archaeal genomes (for a detailed picture of the genome context of all DNA replication genes examined in this study see Additional data file 2). **(a) **The gene encoding Gins15 is linked to the gene coding for PCNA and to the gene for the small subunit of the primase in all crenarchaeal genomes, whereas it is alternatively linked to one of these two genes in most euryarchaeal genomes. **(b) **The gene for the PCNA associates with the genes encoding the small or the large subunit of the DNA primase. It is also frequently linked to the gene encoding TFS and/or to the gene coding for the ADP-ribose pyrophosphatase NudF. **(c) **The gene encoding the MCM helicase is contiguous to the gene for Gins23 and/or to the gene for the beta subunit of the initiation factor aIF-2 in several archaeal genomes. Orthologous genes are indicated in the same color. Each gene is denoted by the name of the protein it encodes (see the key at the bottom). Species or cell lineages that have the same genomic environment are listed and the number of corresponding genomes is given in parentheses. White arrows correspond to additional functionally unrelated genes. Genes are not shown to scale.

Since DNA replication proteins should interact physically and/or functionally in the replication factory, one can expect that genes encoding different DNA replication proteins sometimes co-localize in archaeal genomes, as a blueprint for these interactions. Such DNA replication islands were previously observed in the vicinity of the *Pyrococcus abyssi *chromosomal replication origin (*oriC*), where the gene encoding Cdc6 lies together with those encoding DP1, DP2, RFC-s, and RFC-l [22]; and at the *cdc6-2 *locus in *Sulfolobus solfataricus*, where the genes encoding RFC-s, RFC-l, Cdc6-2, Gins23, and MCM are situated [23,24]. We have detected several new DNA replication islands in our analysis. The association of the genes encoding PCNA, PriS, and Gins15 (hereafter called the PPsG cluster), previously observed by others [14,24], is the most conserved clustering. The full PPsG cluster is not conserved across the entire archaeal domain since the three corresponding genes are adjacent only in crenarchaeal genomes, but the gene encoding Gins15 is contiguous to either the gene for PCNA or the gene for PriS in most euryarchaeal genomes, strongly suggesting that Gins15, PCNA, and PriS functionally associate (Figure 2a). Hence, the genes encoding Gins15 and PCNA are direct neighbors in the four Thermococcales, in two Methanococcales, and in two Methanobacteriales, whereas the genes encoding Gins15 and PriS are adjacent in Methanosarcinales (four species) and in halophilic Archaea (three species). Interestingly, while the gene encoding PCNA is an immediate neighbor of PriS in the PPsG cluster, it co-localizes with the gene encoding the other primase subunit, PriL, in the four Methanosarcinales, in *A. fulgidus*, *Haloarcula marismortui*, and *Halobacterium salinarum *(Figure 2b). In summary, the gene encoding Gins15 is associated with the genes encoding PriS and PCNA (Crenarchaea) or contiguous to one of these two genes (Euryarchaea), whilst the gene coding for PCNA is linked either to the gene encoding PriS (Crenarchaea) or to the gene coding for PriL (Euryarchaea) (Figure 2a,b). This suggests that PCNA could interact with the two primase subunits, whereas Gins15 could interact directly with PCNA and PriS. Finally, the gene encoding Gins23, which has been detected only in Crenarchaea and Thermococcales, neighbors the gene encoding MCM in all these Archaea, except in *P. aerophilum *(Figure 2c).

Altogether, these observations suggest the existence of a core of DNA replication factors, including the PCNA clamp, the DNA primase, the GINS complex, and the helicase MCM, that should be tightly associated with the replication factory during the elongation step of DNA replication. Bell and colleagues [24] have demonstrated by two-hybrid analysis in yeast and immunoprecipitation that the two *Sulfolobus *Gins proteins indeed form a complex that interacts with MCM and the two subunits of the DNA primase. They have suggested that this complex could provide a mechanism to couple the progression of the MCM helicase on the leading strand with priming events on the lagging strand [24]. Our genome context analysis further suggests that PCNA could interact with the GINS complex (via Gins15) and with each of the two subunits of the DNA primase. However, no interaction between PCNA and any of the Gins subunits has been detected by Bell and colleagues [24]. Similarly, no interaction between PCNA and the DNA primase has ever been reported in Archaea, despite the recurrent association of their genes in archaeal genomes. But, it should be noted that the gene for PCNA and the gene for PriS are probably co-transcribed [35], thus strengthening our predictions.

### A specific link between PCNA and DNA primase

We noticed that the gene encoding PCNA is often associated with one or two of the genes coding for the subunits of the DNA primase. This linking is especially conserved since it occurs both in the PPsG cluster and in additional contexts. Hence, the gene for PCNA is adjacent to the gene encoding the large subunit of the DNA primase in *A. fulgidus*, *M. hungatei*, *H. salinarum*, *H. marismortui*, and Methanosarcinales (Figure 2b). Besides the likely association of these two factors at the replication fork, an interesting hypothesis is that it could also reflect the involvement of the archaeal primase in DNA repair, since the PCNA clamp is an accessory factor of many DNA repair proteins. It has been previously suggested that archaeal DNA primase may be involved in DNA repair processes as a translesion DNA polymerase, since most archaeal genomes lack genes encoding DNA polymerases of the X or Y families, which are the major translesion DNA polymerases in bacteria or eukaryotes [36]. The DNA primases from *Pyrococcus furiosus *and *S. solfataricus *are indeed able to synthesize DNA strands *in vitro *(reviewed in [36]) and a translesion synthesis activity has been recently detected in fractions containing the DNA primase in partially purified *P. furiosus *cell extracts [37]. Finally, the catalytic site of the archaeal primase exhibits some structural similarities with the repair DNA polymerase of the X family (reviewed in [36]). Therefore, it is tempting to speculate that PCNA contacts the DNA primase during DNA repair transactions and that the genomic association highlighted in this work is functionally relevant.

### Interactions between DNA replication and DNA repair

In the course of this analysis, we detected many genomic associations of DNA replication genes with genes coding for archaeal homologs of DNA repair/recombination proteins from Eukarya (XPF, RadA, RadB, Mre11, Rad50) or from Bacteria (PolX, RecJ, Endo III, Endo IV, Endo V, UvrABC). We also found associations between genes for DNA replication proteins and specific archaeal proteins that have been characterized biochemically and predicted to be involved in the repair of stalled replication forks by recombination/repair (the helicase Hel308a/Hjm, a RecQ analogue; the nuclease/helicase Hef; and the Holliday junction resolvase Hjc). All these observations suggest that several DNA replication proteins are also involved in base excision repair, in nucleotide excision repair, or in the repair of stalled replication forks. They are described and discussed in Additional data file 3.

### Functional connection of DNA replication, transcription, and DNA repair processes via the TFS and NudF proteins?

We observed an unexpected conserved association between the genes coding for PCNA and TFS. These two genes are neighbors in both crenarchaeal (*P. aerophilum*, *Aeropyrum pernix*) and euryarchaeal genomes (Thermococcales, Methanobacteriales and Methanosarcinales) (Figure 2b). In *P. aerophilum *and *A. pernix*, the gene coding for TFS is located just upstream of the PPsG cluster, whereas it forms a cluster with the genes coding for PCNA and Gins15 in Thermococcales and Methanobacteriales, and with those encoding PCNA and PriL in Methanosarcinales (Figure 2b).

In summary, the gene for PCNA is linked to the gene coding for TFS in 12 out of the 27 analyzed genomes. Although, this gene pairing is not supported by statistical analyses since two genes clusters are frequently conserved across genomes (Additional data file 4), it cannot be a chance occurrence (see below in the Statistical analyses section). Furthermore, it is remarkable that these two genes are associated in both crenarchaeal and euryarchaeal genomes representing four different orders. In our opinion, this conservation pattern indicates that this gene pairing is not coincidental, pointing towards the existence of cross-talk between replication and transcription processes and indicating that TFS and PCNA may be part of this connection. The archaeal protein TFS is homologous to the carboxy-terminal domain of the eukaryotic transcription factor TFIIS and to one of the small subunits of the three eukaryotic RNA polymerases [38]. TFS is also a functional analogue of the bacterial GreA/GreB proteins. When an RNA polymerase is blocked by a DNA lesion, all these proteins can activate an intrinsic 3' to 5' RNase activity of the RNA polymerase, allowing degradation of the mRNA and re-initiation of transcription [39]. It has been shown *in vitro *that misincorporation of non-templated nucleotide is reduced in the presence of archaeal TFS and that TFS helps the elongation complex to bypass a variety of obstacles in front of transcription forks [39]. One possibility, suggested by our genome context analysis, is that TFS recruits DNA repair proteins via PCNA when a DNA replication fork encounters a transcription fork blocked by a DNA lesion. In agreement with a direct role of TFS in controlling genome stability, *M. kandleri*, which is the only archaeon lacking TFS, exhibits a high frequency of gene rearrangement (fusion, splitting) and gene capture, whereas its RNA polymerase has evolved more rapidly than other archaeal RNA polymerases [40].

Interestingly, the gene coding for TFS co-localizes in several euryarchaeal genomes with a gene encoding a protein belonging to the Nudix phosphohydrolase superfamily (Nudix stands for Nucleoside diphosphate linked to another moiety, X). Nudix proteins, which are found in the three domains of life, hydrolyze a wide range of organic pyrophosphates, including nucleoside di- and triphosphates, dinucleoside polyphosphate, and nucleotide sugars; some superfamily members have the ability to degrade damaged nucleotides (reviewed in [41]). We noticed that the Nudix hydrolase encoded by the gene that is arranged in tandem with the gene coding for TFS has been characterized as an ADP-ribose pyrophosphatase in *M. jannaschii *[42]. Therefore, we suggest that every Nudix gene that is linked to a TFS gene in archaeal genomes likely encodes a protein with a similar function (hereafter called NudF protein according to the nomenclature found in [41]). The clustering between the genes encoding TFS and NudF was previously noticed by Dandekar and co-workers [2] (the NudF protein is mentioned by the name 'MutT-like' in this article), who proposed a physical interaction between the two proteins using structural modeling data. The genes encoding NudF and TFS co-localize with those encoding PCNA and PriL in Methanosarcinales, and with those encoding PCNA and Gins15 in Methanobacteriales (Figure 2b). Remarkably, in *M. kandleri*, which does not contain any TFS homolog, the gene for NudF co-localizes with the PCNA gene (Figure 2b). All these observations suggest that, together with TFS, NudF could be associated at the replication forks with the core of proteins previously identified through the PPsG cluster. The role of NudF could be to hydrolyze damaged nucleotides, in order to prevent their incorporation by DNA or RNA polymerases. However, considering that NudF is an ADP-ribose pyrophosphatase [42], an attractive alternative hypothesis is that NudF participates in a network of activities that regulate DNA replication/repair via ADP-ribosylation. In eukaryotes, several DNA replication factors, such as PCNA, primase and DNA polymerases, are indeed poly-ADP-ribosylated in response to DNA damage in order to prevent transcription or replication of damaged DNA [43]. Moreover, transient inhibition of DNA replication following DNA damage has been noticed in *P. abyssi *[44]. In Archaea, poly-ADP-ribosylation like reactions have been reported in *S. solfataricus*, and the chromosomal protein Sso7d, which is restricted to Sulfolobales, has been identified as a putative substrate [45]. Interestingly, Sso7d has been recently shown to promote the repair of thymine dimers *in vitro *after photoinduction [46]. If some archaeal proteins involved in DNA replication or transcription are also inhibited by ADP-ribosylation following DNA damage (something that has to be tested), the role of NudF could be, once DNA damage has been repaired, to facilitate replication and/or transcription restart by metabolizing the free ADP-ribose released during degradation of ADP-ribose polymers.

### Genomic contexts of the *cdc6 *gene suggest specific interactions at the replication origin

**Figure 3:**
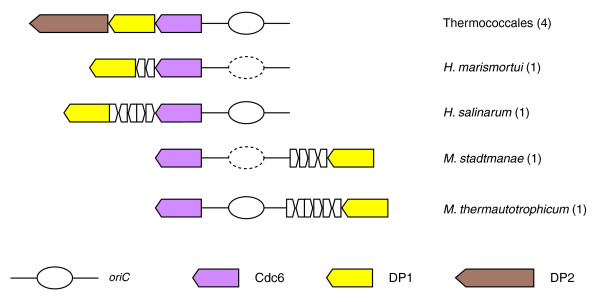
Replication origin is adjacent to *cdc6*, and close to gene for DP1 in several euryarchaeal genomes. Orthologous genes are indicated in the same color. Each gene is denoted by the name of the protein it encodes (see the key at the bottom). The origins of replication (*oriC*) are shown as bubble-shaped replication intermediate sketches; solid lines are used when the origin has been identified experimentally, and broken lines are used when the origin has been predicted with *in silico *analyses. Species or cell lineages that have the same genomic environment are listed and the number of corresponding genomes is given in parentheses. White arrows correspond to additional functionally unrelated genes. Genes are not shown to scale.

Besides the DNA replication genes that belong to the PPsG cluster, the gene that co-localizes more frequently with other DNA replication genes is *cdc6*. Our analysis suggests a loose connection between the initiator protein Cdc6 and the clamp loader RFC, the helicase MCM and DNA polymerases (either B or D), respectively. Hence, the gene encoding Cdc6 is located in the vicinity of the genes encoding RFC-s1 and RFC-l in *P. aerophilum*; RFC-s in *H. salinarum*; MCM and DP2 in *M. maripaludis*; and DP1 in *H. salinarum*, *H. marismortui*, *Methanothermobacter thermautotrophicus*, and *Methanosphaera stadtmanae *(Additional data file 2). Remarkably, all these proteins should be recruited at the replication origin for the initiation of DNA replication. In addition, the genes that are located in the vicinity of the *cdc6 *gene in the genomes of *P. aerophilum*, Halobacteria and methanogens correspond to those that form the replication islands of *Pyrococcus *or *Sulfolobus *(Additional data file 2). Since the gene encoding Cdc6 is frequently associated with a predicted replication origin [22,23,47], co-localization of the *cdc6 *gene with various DNA replication genes in the vicinity of *oriC *could help the recruitment of DNA replication proteins to build new DNA replication factories at the origin of replication. Among the various gene associations of *cdc6 *with other DNA replication genes, the most recurrent is the linkage with the gene encoding the small subunit of PolD. First noticed in *M. thermautotrophicus*, *P. furiosus *and *P. horikoshii *[48], this association turns out to be conserved in all Thermococcales, Halobacteriales, and Methanosarcinales (Figure 3), suggesting that PolD may be recruited by Cdc6 to *oriC *via its small subunit DP1. Interestingly, we recently noticed the presence of an origin recognition box (ORB) and mini-ORB repeats in the gene encoding the DP1 subunit of the four Thermococcales [49]. This suggests that the small subunit of PolD indeed plays a specific role, which remains to be explored in the initiation of DNA replication in Euryarchaeota.

### Identification of new putative DNA replication proteins

We hoped that genome context analysis could help to identify new putative DNA replication proteins in archaeal genomes via the recurrent association of uncharacterized open reading frames to genes encoding already known DNA replication proteins. As previously observed by others [50], and further confirmed by the present analysis, most euryarchaeal genomes (that is, Methanosarcinales, Thermoplasmatales, Halobacteriales, *A. fulgidus*, *M. maripaludis*, and *M. hungatei*) harbor a gene that encodes an OB fold-containing protein without assigned function that is distantly related to the RPA32 subunit of Thermococcales (COG3390). Interestingly, in most euryarchaeal genomes, the gene belonging to COG3390 is arranged in tandem with a gene encoding a RPA41 homolog (which nearly always contains a Zn-finger domain) suggesting that the two gene products functionally associate ([50] and this study; Additional data file 2). Two copies of this RPA41-COG3390 encoding gene cluster are present in Methanosarcinales and Halobacteriales, indicating that the association of the two genes was maintained in both copies after a duplication event that probably occurred before the divergence of these two archaeal lineages. It is tempting to speculate that this RPA32-related protein is a novel single-stranded binding protein that cooperates with RPA in DNA transactions in some euryarchaea.

Another interesting candidate is a protein that we previously identified as PACE12 in a list of proteins from Archaea conserved in Eukarya [51]. Interestingly, the gene encoding PACE12 is located just upstream of the PPsG DNA replication cluster in all Sulfolobales and of the genes encoding MCM and Gins23 in the three *Pyrococcus *species (Figure 2a,c). This suggests that PACE12 could be involved in the network connecting these two clusters. Furthermore, the gene encoding the protein PACE12 co-localizes with the gene encoding DP2 in all Thermoplasmatales (they are both transcribed in the same direction), strengthening the link between PACE12 and DNA replication (Additional data file 2). The PACE12 protein has now been identified as the prototype of a new family of GTPases, the GPN-loop GTPases [52]. Three paralogues of PACE12 are present in eukaryotes and all of them are essential in yeast [53]. One of the human homologs, the protein XAB1 (or MBD*in*), has been shown to be a partner of two proteins: XPA involved in nucleotide excision repair [54] and MBD2, a component of the MeCP1 large protein complex that represses transcription of densely methylated genes [55]. Such observations, together with our genomic context analysis, strengthens the idea that these GTPases are involved in informational mechanisms at the DNA level, possibly related to DNA replication/repair and conserved from Archaea to human.

Finally, our analysis suggests that the archaeal homologs of the bacterial primase DnaG may be involved in DNA replication/repair in Archaea since the gene encoding DnaG is adjacent to the gene encoding PolB3 in the three crenarchaeal lineages investigated and is located in the vicinity of a gene encoding a RPA in almost all Methanosarcinales (Additional data file 2). Furthermore, the gene encoding the archaeal DnaG is located beside the gene encoding PACE12 in *Picrophilus torridus*. The archaeal DnaG-like protein associates with archaeal exosome components in *S. solfataricus *[17] and in *M. thermautotrophicus *[56]. It is usually assumed, therefore, that this protein is not involved in archaeal DNA replication, in agreement with the presence in all Archaea of a eukaryotic-like primase. Our observation nevertheless suggests that DnaG could have diverse roles, one of them being associated with DNA replication or possibly DNA repair.

### Association of DNA replication genes with translation genes

**Figure 4:**
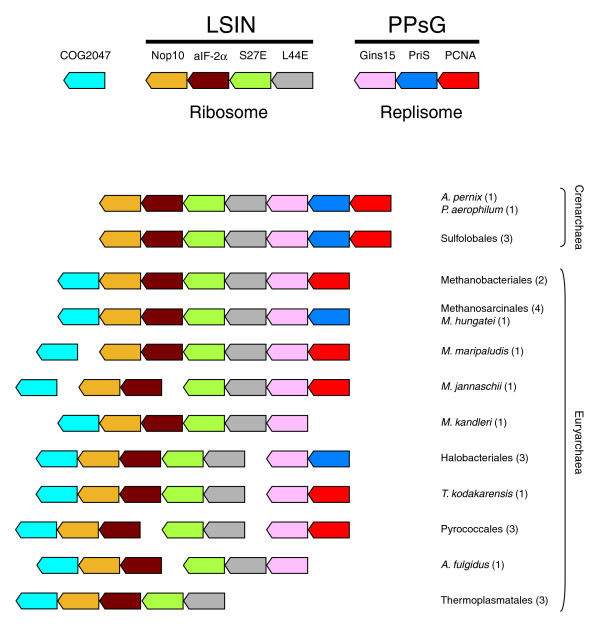
Clustering of DNA replication and ribosome-associated genes in archaeal genomes. Orthologous genes are indicated in the same color. Each gene is denoted by the name of the protein it encodes (see the key at the top). COG2047 encodes an uncharacterized protein of the ATP-grasp superfamily; this COG is absent from Crenarchaea and *N. equitans*. Species or cell lineages that have the same genomic environment are listed and the number of corresponding genomes is given in parentheses. Genes are not shown to scale.

Surprisingly, we found that the DNA replication genes of the PPsG cluster (in crenarchaeal genomes) or its subsets (in euryarchaeal genomes) are frequently contiguous to a set of genes encoding proteins involved in translation. This association forms a supercluster grouping in the same orientation as the genes of the PPsG cluster and a highly conserved cluster of four genes encoding, in order, the ribosomal proteins L44E and S27E, the alpha subunit of the initiation factor aIF-2, and the protein Nop10 (involved in rRNA processing) (hereafter called the LSIN cluster). The complete LSIN cluster is conserved in all Crenarchaea and nearly all Euryarchaea (Figure 4). Surprisingly, despite the nearly systematic conservation of the LSIN cluster in all archaeal lineages, we did not find any publication reporting a direct link between S27E, L44E, aIF-2, and Nop10. A genetic study in yeast pointing toward a role of S27E in rRNA maturation attracted our attention given that Nop10 is involved in this process [57,58]. However, the association of genes coding for S27E, L44E, aIF-2 alpha, and Nop10 is so highly conserved that a link between these four proteins is to be expected. For instance, they could participate in a mechanism coupling ribosome biogenesis to translation, but establishing a functional connection would require further evidence. In euryarchaeal genomes, the gene encoding Nop10 is almost always associated with an additional gene coding for a putative ATPase with no orthologues in crenarchaea and *N. equitans *(COG2047). Therefore, this protein may interact with Nop10, maybe as a regulator given its predicted function.

The genes of the PPsG and LSIN clusters are always organized in the same order and all transcribed in the same direction (Figure 4). This PPsG-LSIN supercluster is complete in all Crenarchaea and nearly complete in Methanobacteriales (with only the gene encoding PriS missing), Methanosarcinales and Methanomicrobiales (with only the gene encoding PCNA missing). Subsets of the PPsG-LSIN supercluster, still consisting of an association between DNA replication and translation protein-encoding genes, are present in *M. kandleri *(G-LSIN), in Methanococcales (PG-LS) and *A. fulgidus *(G-LS). Interestingly, the genes encoding L44E and S27E (LS cluster) are located close to the gene encoding PolB in Thermococcales, whereas the gene encoding Nop10 (N) is close to the gene encoding MCM in *N. equitans*, indicating that the translation proteins encoded by the genes of the LSIN cluster are somehow linked to DNA replication (Additional data file 2).

The archaeal translation initiation factor IF-2 is composed of three subunits, but the three corresponding genes are never adjacent in archaeal genomes. Since the gene encoding the alpha subunit belongs to a conserved operon structure grouping genes encoding DNA replication and translation proteins (Figure 4), we examined the surroundings of the genes encoding the beta and gamma subunits to detect any recurrent gene pairing. Interestingly, the gene for the beta subunit is also associated with DNA replication genes in archaeal genomes since it is adjacent to the gene encoding the replicative helicase MCM (*M. kandleri*, *M. thermautotrophicum*) or forms a cluster together with the genes encoding MCM and Gins23 in the four Thermococcales (Figure 2c). In contrast, the gene coding for the gamma subunit is not linked to DNA replication genes (data not shown). The association of the gene coding for the beta subunit of the initiation factor aIF-2 is not supported by our numerical analysis (Additional data file 4), indicating that this gene pairing may not be significant, although our numerical analysis clearly shows that this association cannot be considered as a chance occurrence (see below). Furthermore, we believe that the presence of DNA replication genes in the vicinity of two of the genes encoding the subunits of the initiation factor aIF-2 is noteworthy. In eukaryotes, eIF-2 is a major target for protein synthesis regulation since its phosphorylation inhibits translation at the initiation step; notably, it has been shown that phosphorylation of the alpha subunit of eIF-2 leads to apoptosis in stress conditions [59]. A recent *in vitro *study has reported that aIF-2 alpha is phosphorylated in a similar fashion to eIF-2 alpha, suggesting the existence of a phosphorylation pathway in the regulation of protein synthesis in Archaea [60]. Our genome context analysis suggests that aIF-2 may associate with both MCM and the gene products of the PPsG cluster via its beta and alpha subunits, respectively (Figures 2c and 4). Given the homology between the translational processes in Archaea and eukaryotes, we speculate that a/eIF-2 could be involved in a mechanism that couples the rate of protein synthesis to the regulation of replication, possibly at the elongation step. Some of the partners of aIF-2 in this process may be among the proteins that are encoded by the genes that associate with the gene coding for aIF-2α in the PPsG-LSIN supercluster.

In the course of performing literature mining regarding these proteins, we focused our attention on S27E since this protein exhibits various extra-ribosomal functions. In human, the gene for this ribosomal protein was originally isolated in a screen for growth factor-induced genes and its product called metallopanstimulin (MPS-1) because it was identified as a metalloprotein expressed in a wide spectrum of proliferating tissues [61]. S27E (MPS-1) is considered as an oncogene and a potential target for cancer therapy because it is highly expressed in actively proliferating cells and cancer cell lines and seems to play a role in progression towards malignancy [62]. Wang and co-workers [62] have recently shown that inactivation of MPS-1 inhibits growth and tumorigenesis and leads to an increase of spontaneous apoptosis in gastric cancer cells. These authors stressed that understanding the mechanism of action of S27E in tumorigenesis "is of paramount interest in the target design for medical intervention in malignant tumor formation" [62]. Interestingly, eukaryotic S27E binds single-stranded as well as double-stranded DNA, with specific binding to the cyclic-AMP responsive element sequence [63]. Several data obtained in eukaryotes indeed suggest that, in addition to its role in the ribosome, S27E may deal with RNA or DNA transaction processes. Hence, S27A mutants in *Arabidopsis thaliana *(S27A is homologous to archaeal S27E) are impaired in the elimination of damaged transcripts after a genotoxic stress, suggesting that S27A is involved in mRNA turnover [64]. Of note, computational analysis showed that S27A from *A. thaliana *exhibits a motif in common with transcriptions factors known to have roles in DNA repair [64]. Thus, S27E may deal with translation as well as ribosome biogenesis, transcription, and DNA repair.

Two main hypotheses can be put forward to explain the genomic association of genes encoding proteins involved in DNA replication and genes coding for proteins involved in translation. First, replication proteins encoded by the PPsG cluster or the translation proteins encoded by the LSIN cluster could have evolved a completely new function, thus harboring two different activities, one in translation and another in replication (moonlighting proteins; for a recent review see [65]); the same property (for example, nucleic acid binding ability) could be used to interact with RNA in a ribosome context and to deal with DNA in a chromosome background. The proteins of the LSIN-PPsG cluster might, therefore, be involved in both translation and DNA replication, independently of any connection between these two processes. A second hypothesis is that the PPsG-LSIN cluster reflects some specific regulatory network coupling DNA replication and translation. The latter hypothesis is more appealing to us than the former since it might be logical to couple ribosome biogenesis and DNA replication to maintain the balance between the amount of DNA and proteins in the cell at different times of the cell cycle. This hypothesis was first proposed by Du and Stillman [66], who reported in yeast that ORC (origin recognition complex) and MCM associate in a complex with proteins involved in ribosome biosynthesis, suggesting potential links between cell proliferation, ribosome biogenesis, and DNA replication. Actually, mounting evidence in eukaryotes points toward a link between ribosome biogenesis and the cell cycle (reviewed in [67]). The existence of a coupling between DNA replication and translation could also possibly explain why the MCM protein of the archaeon *P. abyssi *binds preferentially to the ribosomal operon in stationary phase [49]. Thus, unsuspected links between DNA replication and ribosome biogenesis are emerging piecemeal from biochemical and genetic studies in Archaea and eukaryotes.

### Statistical analysis of genome context supports the cluster of DNA replication and translation genes

**Figure 5:**
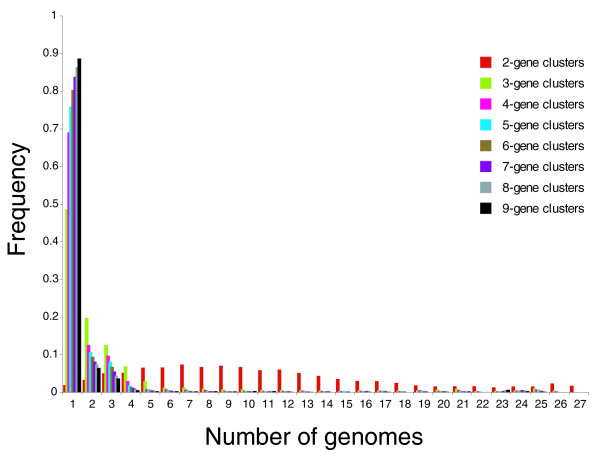
Gene clusters conservation in 27 archaeal genomes. Gene clusters of 2 to 9 genes were searched in 27 archaeal genomes. Two-gene clusters are rather abundant in archaeal genomes; clusters of more than two genes appear mainly in four or fewer genomes.

In order to evaluate the statistical significance of the various genes associations that we have detected in this analysis, we first determined the probability of finding by chance groups of two, three, and so on contiguous genes in a set of 26 randomly shuffled genomes (starting from the genome of *S. acidocaldarius *whose size (2,329 genes) is close to the average size of archaeal genomes). As intuitively expected, we determined that the probability of finding that two neighboring genes in *S. acidocaldarius *are still neighbors in any of the 26 randomized *S. acidocaldarius *genomes is very low (Additional data file 4). For instance, the probabilities of finding that two neighboring genes are still neighbors in two or three randomized genomes is 0.23% and 0.04%, respectively. Accordingly, if two or more genes are located close to each other in the genomes of more than two different species, this cannot be by chance. Two alternatives can be proposed to explain why co-localization of some genes are conserved in several species: these genes were adjacent in the genome of the ancestor of these species and have not yet been separated by chromosome recombination; or there is a selection pressure that favors organisms in which these genes are associated, either by maintaining an association already present in the ancestor of the two genomes or favoring their recurrent association. The distribution of gene clusters in present-day genomes should be the result of a combination of these two alternatives. One can reason that gene clusters maintained only by chance (genes not yet separated by recombination) disappear, on average, more rapidly in the course of evolution than those maintained by selection pressure. In that case, clusters under positive selection pressure should be essentially those present in the highest number of genomes. To perform a quantitative analysis that could be amenable to statistical analysis, we first determined the distribution pattern of gene clusters in our dataset of 27 genomes (see Materials and methods). As shown in Figure 5, we observed that nearly all two-gene clusters (red bar) are present in more than two genomes (up to 27), with a very broad distribution, indicating that genes pairs have been significantly conserved during the evolution of archaeal genomes. In contrast, clusters of three or more genes are much less conserved (most of them being present in from one to four genomes for triplets (green bar) and in one to three genomes for longer clusters). We then calculated a prevalence index based on presence-absence for all gene clusters analyzed, and determined the cumulative index frequency curves for clusters of the same size (see Additional data file 4 for a diagram of curves obtained with clusters of two genes and for clusters of more than two genes). In a traditional statistical approach (one-tailed test), one would consider that a cluster is significant (under positive selection pressure) if its index frequency is located in the portion of the curve corresponding to the 5% less frequent clusters (that is, the very few clusters present in the highest number of genomes), thus strongly deviating from the average of the distribution. The index frequency will hereafter be called the frequency score of this particular cluster. This approach is conservative since it implies that only 5% of the gene clusters in the complete dataset are the result of functional constraints. However, even within a 5% threshold, we found that 13 of the 32 clusters tested in our statistical analysis were supported. These include the supercluster LSIN-PPsG grouping DNA replication and translation genes and most clusters derived from this supercluster (including the PPsG cluster; Additional data file 4). The supercluster LSIN-PPsG itself is highly significant since its frequency score is 2%.

Many potentially interesting gene clusters detected in our analysis (in particular most two-gene clusters) are not statistically supported by a 5% standard threshold. For instance, although the cluster between TFS and PCNA is present in 10 genomes of both Euryarchaea and Crenarchaea (so is probably biologically relevant (see below)), its frequency score is not significant (33%). However, this is also the case for gene associations whose biological relevance has been validated experimentally. For instance, the frequency score of the cluster of genes coding for MCM and Gins23 is not significant (55%) despite the functional relevance of this cluster, as indicated by the work of Bell and colleagues [24]. This again emphasizes that clusters with frequency scores above the 5% threshold are a mixture of clusters maintained only by chance and clusters under selection pressure; there is no easy way to discriminate between them. The best approximation is to consider that clusters under selection pressure are those conserved in genomes from species belonging to different archaeal orders; even more constrained are those conserved across different phyla.

## Conclusion

We have identified, through our genome context analysis of archaeal DNA replication genes, several conserved gene associations that have escaped previous global screening, and from which new functional connections have been inferred. Most of these gene clusters are conserved in distantly related archaeal genomes, indicating that these clusterings are not merely coincidental but probably functionally relevant, and that they should be under selection pressure to optimize functional interactions between the encoded proteins (for instance, via transcriptional co-regulation) and/or to facilitate the formation of specific protein sub-complexes. In particular, we predict that the PCNA clamp, the DNA primase, and the helicase MCM are functionally connected via the GINS complex in the replication factory and that Cdc6 may interact with DP1 at *oriC *for the initiation of DNA replication. We also speculate the existence of cross-talk between DNA replication, DNA repair, and transcription in which PCNA, TFS, and the ADP-pyrophosphatase NudF may be involved. Moreover, we suggest that three proteins without clear functional assignations (an OB-fold containing protein, a recently described new GTPase, DnaG) may take part in informational processes at the DNA level.

Finally, and unexpectedly, we discovered that the genes coding for a particular set of proteins (Gins15, PCNA and/or PriS) are almost systematically arranged in an operon-like structure with a conserved cluster of genes coding for ribosome-related proteins (S27E, L44E, aIF-2α, and Nop10), suggesting the existence of a functional coupling between DNA replication and translation in Archaea. The biological relevance of this association is strongly supported by a statistical analysis of the gene cluster distribution in the 27 archaeal genomes of our dataset. Most of the genes belonging to this particular cluster have eukaryotic homologs but are absent from bacteria; thus, we anticipate that DNA replication and translation may be co-regulated by a mechanism conserved from Archaea to human. The nature of these connections remains to be deciphered but the gene cluster highlighted in this study may be a benchmark for future experimental studies aiming to address this fundamental issue.

## Materials and methods

### Identification of DNA replication genes in archaeal genomes

A list of 12 factors - corresponding to both monomeric and heteromultimeric proteins - likely to be involved in DNA replication was drawn up. This list contains: the initiation factor Cdc6/Orc1; PolB1, PolB2, and PolB3; the small and large subunits of PolD (DP1 and DP2); the helicase MCM; the sliding clamp PCNA; the small and large subunits of the clamp-loader RFC (RFC-s and RFC-l); the small and large subunits of the DNA primase (PriS and PriL); the single-stranded binding protein (RPA or SSB); DNA ligase; the two subunits of Topo VI (Topo VIA and Topo VIB); RNase HII; the flap endonuclease FEN-1; and the two Gins subunits (Gins15 and Gins23) of the GINS complex. The accession numbers of these proteins or protein subunits were retrieved from 27 complete archaeal genomes (*A. pernix*; *P. aerophilum*; the three Sulfolobales, *S. acidocaldarius*, *S. solfataricus*, and *S. tokodaii*; *N. equitans*; *A. fulgidus*; the three Halobacteriales *H. marismortui*, *H. salinarum*, and *Natronomonas pharaonis*; the two Methanobacteriales *M. thermautotrophicus *and *M. stadtmanae*; the two Methanococcales *M. jannaschii *and *M. maripaludis*; *M. kandleri*; the four Methanosarcinales *M. burtonii*, *Methanosarcina acetivorans*, *M. barkeri*, and *M. mazei*; *Methanospirillum hungatei*; the four Thermococcales *P. abyssi*, *P. furiosus*, *P. horikoshii*, and *Thermococcus kodakaraensis*; and the three Thermoplasmatales *Picrophilus torridus*, *Thermoplasma acidophilum*, and *T. volcanium*) by means of BLASTP or PSI-BLAST [68] performed at the NCBI [25] using *P. abyssi *and *S. solfataricus *homologs and, if available, sequences of biochemically characterized proteins as references. All the proteins of the above list were assigned to clusters of orthologous groups (COGs) [69,70] using the COG guess tool from the LBMGE Genomics ToolBox [71] in order to confirm their annotation. Complete archaeal genomes were searched using BLASTP for each class of proteins with various seeds as bait in order to look for misannotated proteins or to uncover overlooked homologs. Finally, BLASTN searches were achieved at the NCBI against the non-redundant archaeal nucleotide sequences database to identify missing open reading frames using closest relative homolog as a query.

### Genome context analysis of DNA replication genes

The genomic context of DNA replication genes were visualized with Genomapper. All genomic contexts were scrutinized manually since the conserved cluster of genes encoding PCNA, PriS and Gins15 was not detected with an automated tool such as STRING [72], likely because sequence similarities are weak between Gins protein family members [24]. In addition, evolutionarily conserved gene neighborhoods turned out to be of valuable importance to identify the archaeal Gins homologs that escaped PSI-BLAST searches. A window encompassing the target gene, the five upstream and five downstream flanking genes was considered during all the genomic environment analysis process. The protein encoded by the genes enclosed in the delimited genomic region were identified using Genome guts [71], assigned to a COG using COG guess, and BLASTP searches against the non-redundant archaeal proteins database were carried out at the NCBI so as to validate their annotation. The surroundings of DNA replication genes that are located on extrachromosomic elements were not inspected since the LBMGE genomes database does not contain archaeal plasmid sequences.

### Statistical analyses

#### Gene cluster conservation in randomized genomes

We chose the *S. acidocaldarius *genome, whose 2,329 genes approximate the average gene content in completely sequenced archaeal genomes, as reference. We generated 26 random genomes as follows: all genes of the *S. acidocaldarius *genome were position-exchanged for another gene chosen randomly from the genome; starting with gene number one and then sequentially applying the same process to all other genes. We then counted the number of times clusters of two to nine genes, present in the genome of *S. acidocaldarius*, remained together in the 26 randomized genomes. For all clusters we calculated a prevalence index based on their presence and absence. That is, every time a gene cluster was indeed present in a randomized genome the prevalence gene index gained one point, otherwise it lost one point. This approach allowed us to calculate the probabilities of having gene clusters by chance only (data not shown). These probabilities were lower than 0.01%, except for clusters of two or three genes in two genomes (see text).

#### Gene cluster conservation in complete archaeal genomes

To establish if the conservation of the gene clusters characterized in this work was statistically significant, we decided to determine the global gene cluster conservation among the 27 archaeal genomes we used for genome context analysis. A genome was chosen randomly, and from this genome a gene was taken randomly. This gene was then BLAST searched (E-value 0.01) against all other 26 genomes. The same BLAST search (E-value 0.01) was performed for its two neighboring genes (the first upstream and the first downstream). Every time the gene appeared with at least one of the same flanking genes in another genome, the prevalence gene index gained one point, otherwise the index lost one point. The whole operation was repeated 10,000 times. We repeated the same process for gene clusters of three to nine genes. We thus ended up with 10,000 prevalence indexes for each size of gene cluster, from which we constructed frequency distributions (examples of these distributions can be found in Additional data file 4). At the same time we determined the prevalence indexes of 32 representative clusters containing DNA replication genes and/or translation genes (indexes are shown in Additional data file 4). We then performed a one-tailed test to settle the significance of our clusters; we simply located the prevalence indexes of our 32 clusters in the frequency distributions (frequency score). The indexes were considered statistically supported when they were present in the 5% or less area of the right part of the distributions (examples can be found in Additional data file 4); this area of the distributions contains those very few clusters highly conserved in archaeal genomes.

## Abbreviations

COG, cluster of orthologous groups; DP1, PolD small subunit; DP2, PolD large subunit; LSIN, L44E S27E aIF-2 alpha Nop10; MPS-1, metallopanstimulin 1; OB, oligonucleotide/oligosaccharide-binding; PACE, proteins of Archaea conserved in Eukarya; Pol, DNA polymerase; PPsG, PCNA PriS Gins15; PriL, DNA primase large subunit; PriS, DNA primase small subunit; RFC-l, replication factor C large subunit; RFC-s, replication factor C small subunit; TFS, transcription factor S; Topo, topoisomerase.

## Authors' contributions

PF initiated the study. JB performed genome context analysis. DC carried out statistical analyses and simulations, and helped to interpret numerical analysis. PF and JB interpreted the data and wrote the paper.

## Additional data files

The following additional data are available. Additional data file 1 contains a table listing the DNA replication factors encoded by archaeal genomes analyzed in this work. Additional data file 2 contains several figures showing the genomic context of all the archaeal DNA replication genes analyzed in this study. Additional data file 3 contains a description of and discussion about genomic associations of DNA replication genes with genes coding for archaeal homologs of DNA repair/recombination proteins. Additional data file 4 contains a table with the prevalence indexes of gene clusters and two sketches illustrating frequency distributions of clusters of two genes and clusters of more than two genes.

## Supplementary Material

Additional data file 1 DNA replication factors encoded by archaeal genomes analyzed in this work. Click here for file

Additional data file 2 Genomic context of all the archaeal DNA replication genes analyzed in this study. Click here for file

Additional data file 3 Description of and discussion about genomic associations of DNA replication genes with genes coding for archaeal homologs of DNA repair/recombination proteins. Click here for file

Additional data file 4 Prevalence indexes of gene clusters and frequency distributions of clusters of two genes and clusters of more than two genes. Click here for file
