# Supplementary material for: Genomic context analysis in Archaea suggests previously unrecognized links between DNA replication and translation
Source: Genome Biol. 2008 Apr 9;9(4):R71. doi: 10.1186/gb-2008-9-4-r71 (PMC2643942; doi:10.1186/gb-2008-9-4-r71)
Supplement: Additional data file 1 — DNA replication factors encoded by archaeal genomes analyzed in this work. [file gb-2008-9-4-r71-S1.pdf]

Additional data file 1

List of archaeal DNA replication factors.

The proteins were identified as described in the Material and methods section. The nomenclature of proteins is indicative as it is not based on phylogenetic analysis. The proteins TVN1466 and TVN1477 encoded by the genome of *Thermoplasma volcanium* are not recognized as Cdc6 homologues (for a recent review, see [20]) but they display similarities with other archaeal Cdc6 homologues and with the Cdc6-related sequence of the ORF1 of the plasmid pTA1 [31]; they are therefore included in the list of Cdc6 homologues and the genome context of their genes is indicated (see additional data file 2). The inteins were identified by means of BLASTP and their presences were confirmed by referring to the New England Biolabs Intein Database [74]. The splitting of the genes coding for the DNA polymerases B from *Nanoarchaeum equitans* and *Methanothermobacter thermautotrophicus* has been reported by Waters and co-workers and Kelman and co-workers, respectively [75, 76]. The sequencing error in the RPA homologue from *Methanothermobacter thermautotrophicus* has been reported by Kelman and co-workers [76]. The nomenclature for RPA and SSB homologues is based on the analysis of Robbins and co-workers [77]. The amino-acid sequences of the RPA and SSB homologues were aligned (data not shown) using CLUSTAL W [78] and MULTALIN [79] to visualize the presence or absence of a zinc-finger domain. Mbur_1377 and Mbur_1735 are annotated as pseudogenes in NCBI genome database so they are indicated between parentheses. Abbreviations: C: Similarity with C-terminal part of MCM (TK0032 shows similarity with PAB0384 and PF1614 but not with MCM); (GC): Genomic context is not shown; FS: Frameshift; LGT: Lateral gene transfer; S: Split; -: Absent; *n: Presence of n intein/homing endonuclease; #: Sequencing error. ¶: CCCH Zn-finger motif; §: CCCC Zn-finger motif.

**Cdc6**			Note
COG1474			
Aeropyrum pernix	APE0152	Cdc6-2	
	APE0475	Cdc6-1	
Archaeoglobus fulgidus	AF0244	Cdc6-1	
	AF0695	Cdc6-2	
Haloarcula marismortui	rrnAC0548		(GC)
	rrnAC1053	Cdc6-1	(GC)
	rrnAC1262	Cdc6-2	(GC)
	rrnAC1568	Cdc6-3	(GC)
	rrnAC1569		(GC)
	rrnAC2385		(GC)
	rrnAC2711	Cdc6-4	
	rrnAC2862	Cdc6-5	
	rrnB0004	Cdc6-6	
	rrnB0063	Cdc6-7	(GC)
	pNG1024 (pNG100)	Cdc6-8	(GC)
	pNG3013 (pNG300)		(GC)
	pNG5027 (pNG500)	Cdc6-9	(GC)
	pNG5122 (pNG500)	Cdc6-10	(GC)
	pNG6174 (pNG600)	Cdc6-11	(GC)
	pNG6184 (pNG600)		(GC)
	pNG7187 (pNG700)	Cdc6-12	(GC)
Halobacterium salinarum	VNG0045C	Cdc6-1	(GC)
	VNG1224G	Cdc6-2	(GC)
	VNG1964H		(GC)
	VNG2271G	Cdc6-4	
	VNG2411G	Cdc6-3	
	VNG7070 (pNRC100)		(GC)
	VNG6091G (pNRC200)		(GC)
	VNG6150G (pNRC200)		(GC)
	VNG6164G (pNRC200)		(GC)
	VNG6187G (pNRC200)		(GC)
	VNG6272G (pNRC200)	Cdc6-5	(GC)
	VNG6363G (pNRC200)	Cdc6-6	(GC)
Methanocaldococcus jannashii	MJ0774	Cdc6	
Methanococcoides burtonii	Mbur_1733	Cdc6-1	
	Mbur_2143	Cdc6-2	
Methanococcus maripaludis	MMP0033	Cdc6	
Methanopyrus kandleri	Not detected		
Methanosarcina acetivorans	MA0001	Cdc6-1	
	MA0085	Cdc6-2	
Methanosarcina barkeri	Mbar_A0976	Cdc6-1	
	Mbar_A1200	Cdc6-2	
	Mbar_B3760 (Plasmid 1)		(GC)

Methanosarcina mazei	MM1314	Cdc6-1	
	MM1378	Cdc6-2	
Methanosphaera stadtmanae	Msp_0001	Cdc6-1	
	Msp_0635	Cdc6-2	
Methanospirillum hungatei	Mhun_0445	Cdc6-2	
	Mhun_2471	Cdc6-1	
Methanothermobacter thermautotrophicus	MTH1412	Cdc6-1	
	MTH1599	Cdc6-2	
	pFV1_p02		(GC)
Nanoarchaeum equitans	NEQ057	Cdc6	
Natronomonas pharaonis	NP0588A	Cdc6-2	
	NP0596A	Cdc6-1	
	NP3096A	Cdc6-3	(GC)
	NP3258A	Cdc6-4	(GC)
	NP6208A	Cdc6-5	(GC)
Picrophilus torridus	PTO1257	Cdc6	
Pyrobaculum aerophilum	PAE0737	Cdc6	
Pyrococcus abyssi	PAB2265	Cdc6	
Pyrococcus furiosus	PF0017	Cdc6	
Pyrococcus horikoshii	PH0124	Cdc6	
Sulfolobus acidocaldarius	Saci_0001	Cdc6-3	
	Saci_0722	Cdc6-1	
	Saci_0903	Cdc6-2	
Sulfolobus solfataricus	SSO0257	Cdc6-1	
	SSO0771	Cdc6-2	
	SSO2184	Cdc6-3	
Sulfolobus tokodaii	ST0305	Cdc6-1	
	ST0471	Cdc6-2	
	ST2165	Cdc6-3	
Thermococcus kodakaraensis	TK1901	Cdc6	
Thermoplasma acidophilum	Ta0451	Cdc6-1	
	Ta0636	Cdc6-2	
	pTA1_01		(GC)
Thermoplasma volcanium	TVN0758	Cdc6-2	
	TVN1044	Cdc6-1	
	TVN1466	Cdc6-4	
	TVN1477	Cdc6-3	

TVN1466 and TVN1477 display strong similarity with the ORF1 from *Thermoplasma acidophilum* plasmid pTA1

**MCM**			
COG1241			
Aeropyrum pernix	APE0188	MCM2	
Archaeoglobus fulgidus	AF0517	MCM2	
Haloarcula marismortui	rrnAC2318	MCM2_1	
	rrnAC2762*1	MCM2_2	
	pNG3053	MCM2_3	(GC)
Halobacterium salinarum	VNG2181G*1	MCM2	
Methanocaldococcus jannashii	MJ0363	MCM2_1	
	MJ0961	MCM2_2	
	MJ1489	MCM2_3	
	MJECL13	MCM2_4	(GC)
Methanococcoides burtonii	Mbur_2432	MCM2	
Methanococcus maripaludis	MMP0030	MCM2_1	
	MMP0470	MCM2_2	
	MMP0748	MCM2_3	
	MMP1024	MCM2_4	
Methanopyrus kandleri	MK0965	MCM2_1	
	MK1120	MCM2_2	
Methanosarcina acetivorans	MA0681	MCM2_1	
	MA3829	MCM2_2	
Methanosarcina barkeri	Mbar_A1593	MCM2	
Methanosarcina mazei	MM1836	MCM2	
Methanosphaera stadtmanae	Msp_0362	MCM2	
Methanospirillum hungatei	Mhun_0985	MCM2	
Methanothermobacter thermautotrophicus	MTH1770	MCM2	
Nanoarchaeum equitans	NEQ282	MCM2	
Natronomonas pharaonis	NP3888A	MCM2_2	
	NP5106A*1	MCM2_1	
Picrophilus torridus	PTO1217	MCM2	
Pyrobaculum aerophilum	PAE0901	MCM2	
Pyrococcus abyssi	PAB2373*2	MCM2	
	PAB0384 (MCM)		
Pyrococcus furiosus	PF0482*1	MCM2	
	PF1614 (MCM)		
Pyrococcus horikoshii	PH0606*2	MCM2	
Sulfolobus acidocaldarius	Saci_0900	MCM2	
Sulfolobus solfataricus	SSO0774	MCM2	
Sulfolobus tokodaii	ST0467	MCM2	
Thermococcus kodakaraensis	TK0096	MCM2_2	
	TK1361	MCM2_3	
	TK1620*2	MCM2_1	
	TK0032 (MCM)		
Thermoplasma acidophilum	Ta0799	MCM2	
Thermoplasma volcanium	TVN1032	MCM2	

*n: Presence of n intein/homing endonuclease
(MCM): Similarity with C-terminal part of MCM.
TK0032 shows similarity with PAB0384 and
PF1614 but not with MCM.

**GINS**	Gins15	Gins23
	COG1711	
Aeropyrum pernix	APE0437	APE0187
Archaeoglobus fulgidus	AF1332	-
Haloarcula marismortui	rrnAC0293	-
Halobacterium salinarum	VNG1471C	-
Methanocaldococcus jannashii	MJ0248	-
Methnococcoides burtonii	Mbur_1393	-
Methanococcus maripaludis	MMP1710	-
Methanopyrus kandleri	MK0416	-
Methanosarcina acetivorans	MA0647	-
Methanosarcina barkeri	Mbar_A1570	-
Methanosarcina mazei	MM1810	-
Methanosphaera stadtmanae	Msp_1530	-
Methanospirillum hungatei	Mhun_1131	-
Methanothermobacter thermautotrophicus	MTH1311	-
Nanoarchaeum equitans	NEQ419	-
Natrosomonas pharaonis	NP3992A	-
Picrophilus torridus	PTO0266	-
Pyrobaculum aerophilum	PAE3035	PAE0965
Pyrococcus abyssi	PAB1466	PAB0956
Pyrococcus furiosus	PF0982	PF0483
Pyrococcus horikoshii	PH0666	PH0610
		PH1177 (GC)
Sulfolobus acidocaldarius	Saci_1278	Saci_0901
Sulfolobus solfataricus	SSO1049	SSO0772
Sulfolobus tokodaii	ST0942	ST0469
Thermococcus kodakaraensis	TK0536	TK1619
Thermoplasma acidophilum	Ta1042	-
Thermoplasma volcanium	TVN0553	-

-: Absent

**DNA primase**	Primase small subunit PriS	Primase large subunit PriL
	COG1467	COG2219
Aeropyrum pernix	APE0438	APE0667
Archaeoglobus fulgidus	AF0742	AF0336
Haloarcula marismortui	rrnAC0292	rrnAC2844
Halobacterium salinarum	VNG1470G	VNG2254C
Methanocaldococcus jannashii	MJ0839	MJ0701
Methanococcoides burtonii	Mbur_1392	Mbur_2192
Methanococcus maripaludis	MMP0071	MMP0009
Methanopyrus kandleri	MK0586	MK1394
Methanosarcina acetivorans	MA0648	MA0109
Methanosarcina barkeri	Mbar_A1571	Mbar_A0880
Methanosarcina mazei	MM1811	MM1396
Methanosphaera stadtmanae	Msp_0085	Msp_0084
Methanospirillum hungatei	Mhun_1132	Mhun_1489
Methanothermobacter thermautotrophicus	MTH585	MTH586
Nanoarchaeum equitans	NEQ395	
Natronomonas pharaonis	NP3990A	NP0686A
Picrophilus torridus	PTO0802	PTO0982
Pyrobaculum aerophilum	PAE3036	PAE2238
Pyrococcus abyssi	PAB2236	PAB2235
Pyrococcus furiosus	PF0110	PF0111
Pyrococcus horikoshii	PH0195	PH0196
Sulfolobus acidocaldarius	Saci_1279	Saci_1542
Sulfolobus solfataricus	SSO1048	SSO0557
Sulfolobus tokodaii	ST0943	ST1431
Thermococcus kodakaraensis	TK1791	TK1790
Thermoplasma acidophilum	Ta0975	Ta1038
Thermoplasma volcanium	TVN1121	TVN0557

**PCNA**	Euryarchaea		Crenarchaea	
COG0592	Nanoarchaea			
	PCNA	PCNA1	PCNA2	PCNA3
Aeropyrum pernix		APE0162	APE0441	APE2182
Archaeoglobus fulgidus	AF0335			
Haloarcula marismortui	rrnAC2851			
Halobacterium salinarum	VNG2256G			
Methanocaldococcus jannashii	MJ0247			
Methanococcoides burtonii	Mbur_2193			
Methanococcus maripaludis	MMP1711			
Methanopyrus kandleri	MK1030			
Methanosarcina acetivorans	MA0110			
Methanosarcina barkeri	Mbar_A0879			
Methanosarcina mazei	MM1397			
Methanosphaera stadtmanae	Msp_1531			
Methanospirillum hungatei	Mhun_1488			
Methanothermobacter thermautotrophicus	MTH1312			
Nanoarchaeum equitans	NEQ537			
Natronomonas pharaonis	NP0554A			
Picrophilus torridus	PTO1316			
Pyrobaculum aerophilum		PAE0720	PAE3038	
Pyrococcus abyssi	PAB1465			
Pyrococcus furiosus	PF0983			
Pyrococcus horikoshii	PH0665			
Sulfolobus acidocaldarius		Saci_0817	Saci_1280	Saci_0826
Sulfolobus solfataricus		SSO0397	SSO1047	SSO0405
Sulfolobus tokodaii		ST0397	ST0944	ST0387
Thermococcus kodakaraensis	TK0535			
	TK0582			
Thermoplasma acidophilum	Ta0917			
Thermoplasma volcanium	TVN1062			

**DNA polymerase B**	DNA polymerase B1<br>PolB1	DNA Polymerase B3<br>PolB3	DNA Polymerase B2<br>PolB2
COG0417			
Aeropyrum pernix	APE0099	APE2098	APE2229
Archaeoglobus fulgidus	-	AF0497	AF0693m
Haloarcula marismortui	-	rrnAC1831*1	pNG6176<br>(pNG600) (GC)
Halobacterium salinarum	-	VNG0521G	VNG6362G<br>(pNRC200) (GC)
Methanocaldococcus jannashii	-	MJ0885*2	
Methanococcoides burtonii	-	Mbur_1688	Mbur_0564 (GC)
			Mbur_0569 (GC)
			Mbur_0577 (GC)
Methanococcus maripaludis	-	MMP0380	
Methanopyrus kandleri	-	MK1039	
Methanosarcina acetivorans	-	MA0885	
Methanosarcina barkeri	-	Mbar_A1777	
Methanosarcina mazei	-	MM2004	MM3253
Methanosphaera stadtmanae	-	Msp_1507	
	-	Msp_1281	
Methanospirillum hungatei	-	Mhun_1156	Mhun_0839
Methanothermobacter thermautotrophicus	-	MTH208 S	
		MTH1208 S	
Nanoarchaeum equitans	-	NEQ068*1 S	
		NEQ528*1 S	
Natronomonas pharaonis	-	NP1508A*2	
Picrophilus torridus	-	PTO0128	
Pyrobaculum aerophilum	PAE2180	PAE2109	PAE1113
Pyrococcus abyssi	-	PAB1128	
Pyrococcus furiosus	-	PF0212	
Pyrococcus horikoshii	-	PH1947*1	
Sulfolobus acidocaldarius	Saci_1537	Saci_0074	-
Sulfolobus solfataricus	SSO0552	SSO0081	SSO1459<br>SSO8124 FS
Sulfolobus tokodaii	ST1426	ST2076	ST1680<br>STS189 FS
Thermococcus kodakaraensis	-	TK0001*2	
Thermoplasma acidophilum	-	Ta0907	Ta0450
Thermoplasma volcanium	-	TVN0823	TVN1045

S=split
*n: Presence of n intein/homing endonuclease
FS: Frameshift

**DNA polymerase D**	DNA polymerase D<br>Small subunit<br>DP1	DNA polymerase D<br>Large subunit<br>DP2
	COG1311	COG1933
Aeropyrum pernix	-	-
Archaeoglobus fulgidus	AF1790	AF1722
Haloarcula marismortui	rrnAC2714	rrnAC2691*1
Halobacterium salinarum	VNG2417G	VNG2338G*1
Methanocaldococcus jannashii	MJ0702	MJ1630
Methanococcoides burtonii	(Mbur_1735)	Mbur_2423
Methanococcus maripaludis	MMP0008	MMP0026
Methanopyrus kandleri	MK1583	MK1650
Methanosarcina acetivorans	MA0037	MA4552
Methanosarcina barkeri	Mbar_A1005	Mbar_A0899
Methanosarcina mazei	MM1345	MM1246
Methanosphaera stadtmanae	Msp_1584	Msp_0255
Methanospirillum hungatei	Mhun_2412	Mhun_2435*1
Methanothermobacter thermautotrophicus	MTH1405	MTH1536
Nanoarchaeum equitans	NEQ240	NEQ420
Natronomonas pharaonis	NP0482A	NP0476A
Picrophilus torridus	PTO0469	PTO0558
Pyrobaculum aerophilum	-	-
Pyrococcus abyssi	PAB2266	PAB2404*1
Pyrococcus furiosus	PF0018	PF0019
Pyrococcus horikoshii	PH0123	PH0121*1
Sulfolobus acidocaldarius	-	-
Sulfolobus solfataricus	-	-
Sulfolobus tokodaii	-	-
Thermococcus kodakaraensis	TK1902	TK1903*1
Thermoplasma acidophilum	Ta0222	Ta0036
Thermoplasma volcanium	TVN1372	TVN0035

*n: Presence of n intein/homing endonuclease
(Pseudogene)

**Replication factor C (RFC)**	RFC<br>small subunit<br>(RFC-s)		RFC<br>large subunit<br>(RFC-l)	
	COG0470		COG0470	
Aeropyrum pernix	APE1522	RFC-s	APE1524	RFC-l
Archaeoglobus fulgidus	AF2060	RFC-s	AF1195	RFC-l
Haloarcula marismortui	rrnAC2487	RFC-s2	rrnAC1744	RFC-l
	rrnAC2565	RFC-s1		
Halobacterium salinarum	VNG2240G	RFC-s2	VNG1622G	RFC-l
	VNG2280G	RFC-s1		
Methanocaldococcus jannashii	MJ1422*3	RFC-s	MJ0884	RFC-l
Methanococcoides burtonii	Mbur_1988	RFC-s2	Mbur_2242	RFC-l
	(Mbur_1377)	(RFC-s1)		
Methanococcus maripaludis	MMP0427	RFC-s	MMP0322	RFC-l
Methanopyrus kandleri	MK0006*1	RFC-s	MK0005	RFC-l
Methanosarcina acetivorans	MA0140	RFC-s2	MA1810	RFC-l
	MA0669	RFC-s1		
Methanosarcina barkeri	Mbar_A1582	RFC-s1	Mbar_A2075	RFC-l
	Mbar_A0850	RFC-s2		
Methanosarcina mazei	MM1426	RFC-s2	MM0253	RFC-l
	MM1821	RFC-s1		
Methanosphaera stadtmanae	Msp_0413	RFC-s	Msp_0414	RFC-l
Methanospirillum hungatei	Mhun_0833	RFC-s2	Mhun_0916	RFC-l
	Mhun_0994	RFC-s1		
Methanothermobacter thermautotrophicus	MTH241	RFC-s	MTH240	RFC-l
Nanoarchaeum equitans	NEQ170	RFC-s	NEQ430	RFC-l
Natronomonas pharaonis	NP0900A	RFC-s1	NP1782A	RFC-l
	NP1068A	RFC-s2		
Picrophilus torridus	PTO0587	RFC-s	PTO0873	RFC-l
Pyrobaculum aerophilum	PAE0734	RFC-s1	PAE0735	RFC-l
	PAE1646	RFC-s2		
Pyrococcus abyssi	PAB0068*2	RFC-s	PAB0069	RFC-l
Pyrococcus furiosus	PF0093*1	RFC-s	PF0092	RFC-l
Pyrococcus horikoshii	PH0112*1	RFC-s	PH0113	RFC-l
Sulfolobus acidocaldarius	Saci_0907	RFC-s	Saci_0906	RFC-l
Sulfolobus solfataricus	SSO0768	RFC-s	SSO0769	RFC-l

Sulfolobus tokodaii	ST0475	RFC-s	ST0473	RFC-l
Thermococcus kodakaraensis	TK2218*1	RFC-s	TK2219	RFC-l
Thermoplasma acidophilum	Ta1500	RFC-s	Ta1285	RFC-l
Thermoplasma volcanium	TVN1491	RFC-s	TVN0534	RFC-l

*n: Presence of n intein/homing endonuclease
(Pseudogene)

Single-stranded DNA binding protein	RPA		SSB	
Aeropyrum pernix	-		APE1323	SSB
Archaeoglobus fulgidus	AF0382	RPA1	-	
	AF0780 ¶	RPA2		
Haloarcula marismortui	rrnAC1133 ¶	RPA2	-	
	rrnAC2302	RPA1		
	rrnAC2744 ¶	RPA3		
Halobacterium salinarum	VNG0133G	RPA1	-	
	VNG1255C ¶	RPA2		
	VNG2160C ¶	RPA3		
	VNG6403H (pNRC200)	RPA4		
	VNG7134/ VNGH1529 (pNRC100)	RPA5		
Methanocaldococcus jannashii	MJ1159 §	RPA	-	
Methanococcoides burtonii	Mbur_1362 ¶	RPA2		
	Mbur_1374 ¶	RPA3		
	Mbur_1764	RPA1		
Methanococcus maripaludis	MMP0122	RPA1	-	
	MMP1032 §	RPA2		
Methanopyrus kandleri	MK1441 ¶	RPA	-	
Methanosarcina acetivorans	MA0590 ¶	RPA3	-	
	MA3019 ¶	RPA2		
	MA4645	RPA1		
Methanosarcina barkeri	Mbar_A0963	RPA1	-	
	Mbar_A1433 ¶	RPA3		
	Mbar_A2026 ¶	RPA2		
Methanosarcina mazei	MM0293 ¶	RPA2	-	
	MM1299	RPA1		
	MM1751 ¶	RPA3		
Methanosphaera stadtmanae	Msp_1487 §	RPA		
Methanospirillum hungatei	Mhun_2542 ¶	RPA2		
	Mhun_2941	RPA1		
Methanothermobacter thermautotrophicus #	MTH1385	RPA	-	
	MTH1384 §			

Nanoarchaeum equitans	NEQ199	RPA	-	
Natronomonas pharaonis	NP0314A	RPA1		
	NP1282A ¶	RPA3		
	NP3960A ¶	RPA2		
Picrophilus torridus	PTO1419 ¶	RPA	PTO0673	SSB
Pyrobaculum aerophilum	-		Not detected	
Pyrococcus abyssi	PAB2163 ¶	RPA41	-	
	PAB2164	RPA14		
	PAB2165	RPA32		
Pyrococcus furiosus	PF2020 ¶	RPA41	-	
	PF2019	RPA14		
	PF2018	RPA32		
Pyrococcus horikoshii	PH1893 ¶	RPA41	-	
	PH1893.1n	RPA14		
	PH1894	RPA32		
Sulfolobus acidocaldarius	-		Saci_0975	SSB
Sulfolobus solfataricus	-		SSO2364	SSB
Sulfolobus tokodaii	-		ST0503	SSB
Thermococcus kodakaraensis	TK1961 ¶	RPA41	-	
	TK1960	RPA14		
	TK1959	RPA32		
Thermoplasma acidophilum	Ta0387 ¶	RPA	Ta1149	SSB
Thermoplasma volcanium	TVN1185 ¶	RPA	TVN1236	SSB

**DNA ligase**		Note
COG1793		
Aeropyrum pernix	APE1094	
Archaeoglobus fulgidus	AF0623	
	AF1725	LGT
Haloarcula marismortui	rrnAC0463	
Halobacterium salinarum	VNG0881G	
Methanocaldococcus jannashii	MJ0171	
Methanococcoides burtonii	Mbur_1088	
Methanococcus maripaludis	MMP0970	
Methanopyrus kandleri	MK0999	
Methanosarcina acetivorans	MA0728	
	MA2571	
	MA3428	LGT
Methanosarcina barkeri	Mbar_A1899	
	Mbar_A1643	
	Mbar_A2115	LGT
Methanosarcina mazei	MM1895	
	MM2714	
	MM0209	LGT
Methanosphaera stadtmanae	Msp_0258	
Methanospirillum hungatei	Mhun_2882	
Methanothermobacter thermautotrophicus	MTH1580	
Nanoarchaeum equitans	NEQ509	
Natronomonas pharaonis	NP3474A	
Picrophilus torridus	PTO0672	
Pyrobaculum aerophilum	PAE0833	
Pyrococcus abyssi	PAB2002	
Pyrococcus furiosus	PF1635	
Pyrococcus horikoshii	PH1622	
Sulfolobus acidocaldarius	Saci_0788	
Sulfolobus solfataricus	SSO0189	
Sulfolobus tokodaii	ST0223	
Thermococcus kodakaraensis	TK2140	
Thermoplasma acidophilum	Ta1148	
Thermoplasma volcanium	TVN1237	

LGT: Lateral gene transfer

**Flap endonuclease 1 (FEN-1)**	FEN-1
COG0258	
Aeropyrum pernix	APE0115
Archaeoglobus fulgidus	AF0264
Haloarcula marismortui	rrnAC0032
Halobacterium salinarum	VNG1359G
Methanocaldococcus jannashii	MJ1444
Methanococcoides burtonii	Mbur_1913
Methanococcus maripaludis	MMP1313
Methanopyrus kandleri	MK0566
Methanosarcina acetivorans	MA4004
Methanosarcina barkeri	Mbar_A1212
Methanosarcina mazei	MM0906
Methanosphaera stadtmanae	Msp_1086
Methanospirillum hungatei	Mhun_2521
Methanothermobacter thermautotrophicus	MTH1633
Nanoarchaeum equitans	NEQ088
Natronomonas pharaonis	NP3784A
Picrophilus torridus	PTO0228
Pyrobaculum aerophilum	PAE0698
Pyrococcus abyssi	PAB1877
Pyrococcus furiosus	PF1414
Pyrococcus horikoshii	PH1415
Sulfolobus acidocaldarius	Saci_0775
Sulfolobus solfataricus	SSO0179
Sulfolobus tokodaii	ST0210
Thermococcus kodakaraensis	TK1281
Thermoplasma acidophilum	Ta1035
Thermoplasma volcanium	TVN0560

**Ribonuclease HII (RNase HII)**	RNase HII
COG0164	
Aeropyrum pernix	APE0496
Archaeoglobus fulgidus	AF0621
Haloarcula marismortui	rrnAC3216
Halobacterium salinarum	VNG1984G
Methanocaldococcus jannashii	MJ0135
Methanococcoides burtonii	Mbur_0450
Methanococcus maripaludis	MMP1374
Methanopyrus kandleri	MK0186
Methanosarcina acetivorans	MA1959
Methanosarcina barkeri	Mbar_A2753
Methanosarcina mazei	MM2814
Methanosphaera stadtmanae	Msp_1395
Methanospirillum hungatei	Mhun_1216
Methanothermobacter thermautotrophicus	MTH1023
Nanoarchaeum equitans	NEQ063
Natronomonas pharaonis	NP2392A
Picrophilus torridus	PTO1263
Pyrobaculum aerophilum	PAE1216
Pyrococcus abyssi	PAB0352
Pyrococcus furiosus	PF1781
Pyrococcus horikoshii	PH1650
Sulfolobus acidocaldarius	Saci_0958
Sulfolobus solfataricus	SSO2384
Sulfolobus tokodaii	ST0519
Thermococcus kodakaraensis	TK0805
Thermoplasma acidophilum	Ta1458
Thermoplasma volcanium	TVN0133

**Topoisomerase VI**	DNA topoisomerase VI Subunit A	DNA topoisomerase VI Subunit B
	COG1697	COG1389
Aeropyrum pernix	APE0703	APE0706
Archaeoglobus fulgidus	AF0940	AF0652
Haloarcula marismortui	rrnAC0459	rrnAC0457
Halobacterium salinarum	VNG0884G	VNG0885G
Methanocaldococcus jannashii	MJ0369	MJ1028
Methanococcoides burtonii	Mbur_1202	Mbur_1203
Methanococcus maripaludis	MMP1437	MMP0989
Methanopyrus kandleri	MK0512	MK0921
Methanosarcina acetivorans	MA1586	MA1587
Methanosarcina barkeri	Mbar_A2806	Mbar_A2807
Methanosarcina mazei	MM2418	MM2417
Methanosphaera stadtmanae	Msp_1340	Msp_1339
Methanospirillum hungatei	Mhun_2920	Mhun_2919
Methanothermobacter thermautotrophicus	MTH1008	MTH1007
Nanoarchaeum equitans	NEQ542	NEQ144
Natronomonas pharaonis	NP3480A	NP3482A
Picrophilus torridus	-	
Pyrobaculum aerophilum	PAE2219	PAE2217
Pyrococcus abyssi	PAB2411	PAB0407
Pyrococcus furiosus	PF1578	PF1579
Pyrococcus horikoshii	PH1563	PH1564
Sulfolobus acidocaldarius	Saci_1314	Saci_1315
Sulfolobus solfataricus	SSO0969	SSO0968
Sulfolobus tokodaii	ST1295	ST1294
Thermococcus kodakaraensis	TK0798	TK0799
Thermoplasma acidophilum	-	
Thermoplasma volcanium	-	

-: Absent
