# Supplementary material for: Genomic context analysis in Archaea suggests previously unrecognized links between DNA replication and translation
Source: Genome Biol. 2008 Apr 9;9(4):R71. doi: 10.1186/gb-2008-9-4-r71 (PMC2643942; doi:10.1186/gb-2008-9-4-r71)
Supplement: Additional data file 2 — Genomic context of all the archaeal DNA replication genes analyzed in this study. [file gb-2008-9-4-r71-S2.pdf]

Additional data file 2

Genomic context of DNA replication genes in archaeal genomes.

Orthologous genes that encode archaeal DNA replication factors (see Material and methods section in the main text for the list) or genes that belong to the supercluster revealed by this study are colored in a same fashion and are denoted by the names of the protein they respectively encode. Neighboring genes belonging to the COG class J (translation, ribosomal structure, and biogenesis), K (transcription) and L (replication, recombination, and repair) are colored in grey, light blue, and yellow respectively in order to distinguish them easily from other categories of genes. Pseudogenes are indicated by a broken lined edged arrow. The origins of replication are shown as bubble-shaped replication intermediates sketches; solid lanes are used when the origin has been identified experimentally, broken lines are employed when the origin has been predicted with *in silico* analyses. The gene neighborhood that prevails among a group of closely related archaea is shown except when an association between DNA replication and translation or transcription genes occurring in one but not all genomes has been noticed. Small triangle-shaped genes sketches enclosed between parentheses correspond to a slight variation observed in one or a few organisms compared with the displayed genomic context. For sake of clarity, some genes are not shown; in this case a solid line is drawn and the number of hidden genes is mentioned. Arrows are oriented according to the direction of transcription. Genes are not shown to scale. Abbreviations: *Hma: Haloarcula marismortui*; *Hsa: Halobacterium salinarum*; *Mac: Methanosarcina acetivorans*; *Mba: Methanosarcina barkeri*; *Mbu: Methanococcoides burtonii*; *Mja: Methanocaldococcus jannaschii*; *Mma: Methanosarcina. mazei*; *Mmp: Methanococcus maripaludis*; *Msp: Methanosphaera stadtmanae*; *Mth: Methanothermobacter thermautotrophicus*; *Nph: Natronomonas pharaonis*; *Pab: Pyroccoccus abyssi*; *Pfu: Pyrococcus furiosus*; *Pho:*

Pyrococcus horikoshii; *Pto: Picrophilus torridus*; Pyr.: Pyrococcales; *Saci: Sulfolobus acidocaldarius*; *Sso: Sulfolobus solfataricus*; *Sto: Sulfolobus tokodaii*; *Tac: Thermoplasma acidophilum*; *Tko: Thermococcus kodakaraensis*; *Tvo: Thermoplasma volcanium.*

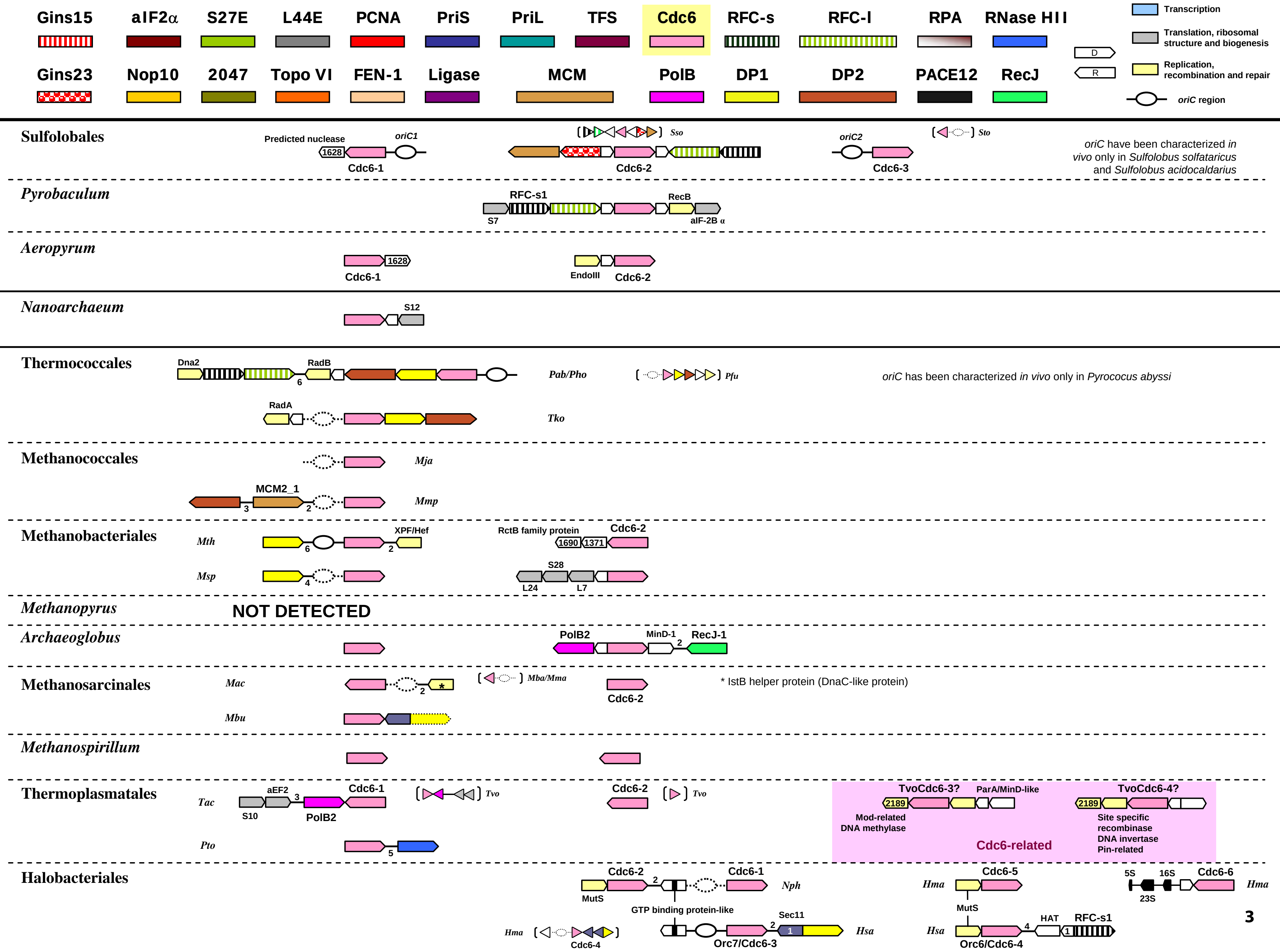

Gins15
aIF2α
S27E
L44E
PCNA
PriS
PriL
TFS
Cdc6
RFC-s
RFC-l
RPA
RNase HII
Gins23
Nop10
2047
Topo VI
FEN-1
Ligase
MCM
PolB
DP1
DP2
PACE12
RecJ
D
R
Transcription
Translation, ribosomal structure and biogenesis
Replication, recombination and repair
oriC region
Sulfolobales
Predicted nuclease
1628
oriC1
Cdc6-1
Sso
Cdc6-2
oriC2
Sto
Cdc6-3
oriC have been characterized in vivo only in Sulfolobus solfataricus and Sulfolobus acidocaldarius
Pyrobaculum
RFC-s1
RecB
S7
aIF-2B α
Aeropyrum
1628
Cdc6-1
EndoIII
Cdc6-2
Nanoarchaeum
S12
Thermococcales
Dna2
RadB
6
Pab/Pho
Pfu
oriC has been characterized in vivo only in Pyrococus abyssi
RadA
Tko
Methanococcales
Mja
MCM2_1
3
2
Mmp
Methanobacteriales
Mth
6
XPF/Hef
2
RctB family protein
1690
1371
Cdc6-2
Msp
4
S28
L24
L7
Methanopyrus
NOT DETECTED
Archaeoglobus
PolB2
MinD-1
2
RecJ-1
Methanosarcinales
Mac
2
*
Mba/Mma
Cdc6-2
* IstB helper protein (DnaC-like protein)
Mbu
Methanospirillum
Thermoplasmatales
Tac
aEF2
3
S10
PolB2
Cdc6-1
Tvo
Cdc6-2
Tvo
TvoCdc6-3?
ParA/MinD-like
2189
Mod-related DNA methylase
TvoCdc6-4?
2189
Site specific recombinase DNA invertase Pin-related
Cdc6-related
Pto
5
Halobacteriales
Cdc6-2
2
MutS
Cdc6-1
Nph
GTP binding protein-like
Hma
Cdc6-5
MutS
5S
16S
23S
Cdc6-6
Hma
Hma
Cdc6-4
Sec11
2
1
Orc7/Cdc6-3
Hsa
Hsa
4
HAT
RFC-s1
1
Orc6/Cdc6-4

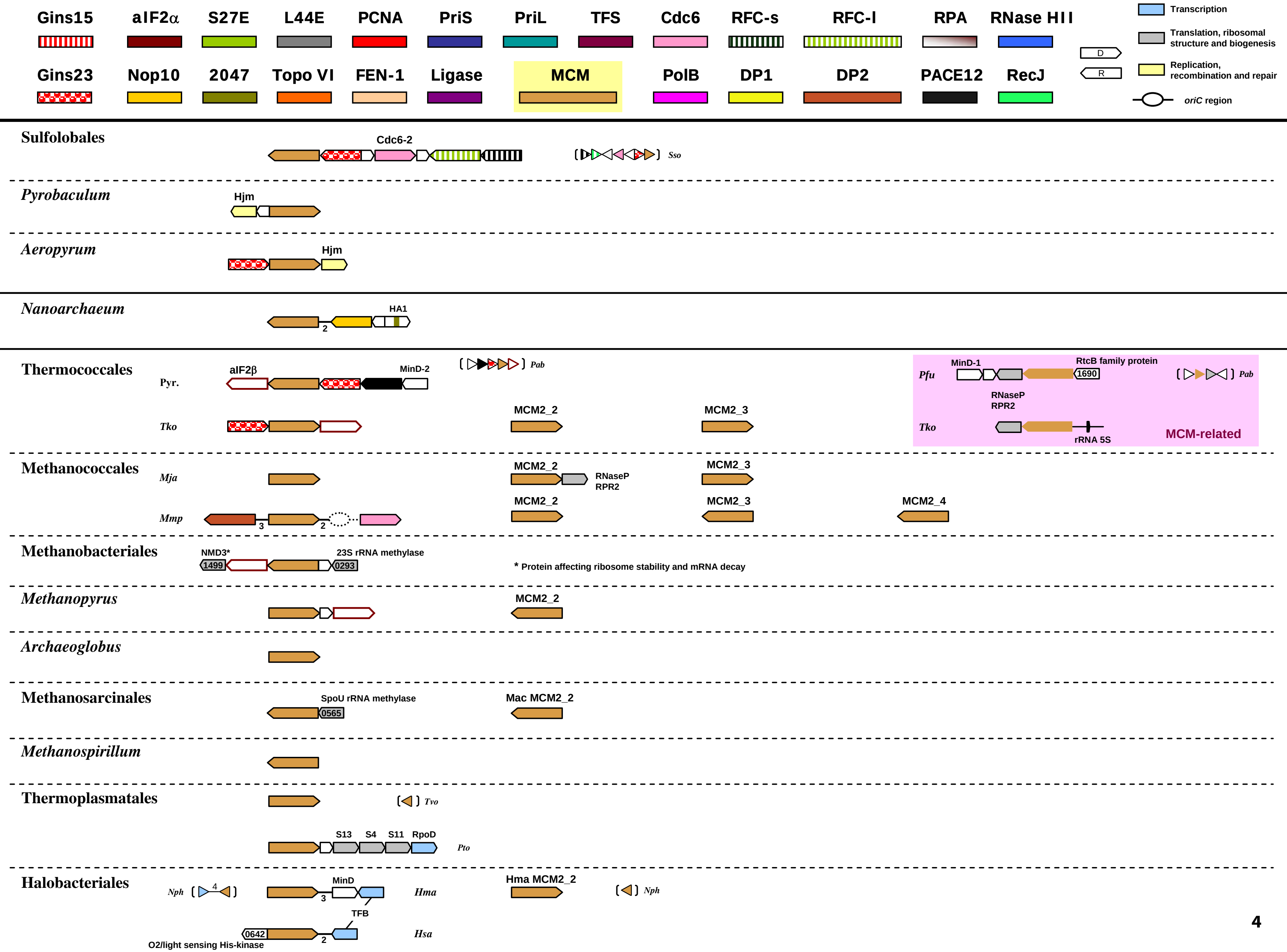

Gins15
aIF2α
S27E
L44E
PCNA
PriS
PriL
TFS
Cdc6
RFC-s
RFC-l
RPA
RNase HII
Gins23
Nop10
2047
Topo VI
FEN-1
Ligase
MCM
PolB
DP1
DP2
PACE12
RecJ
D
R
Transcription
Translation, ribosomal structure and biogenesis
Replication, recombination and repair
oriC region
Sulfolobales
Cdc6-2
Sso
Pyrobaculum
Hjm
Aeropyrum
Hjm
Nanoarchaeum
HA1
2
Thermococcales
Pyr.
aIF2β
MinD-2
Pab
Tko
MCM2_2
MCM2_3
Pfu
MinD-1
RtcB family protein
1690
Pab
RNaseP RPR2
Tko
rRNA 5S
MCM-related
Methanococcales
Mja
MCM2_2
RNaseP RPR2
MCM2_3
Mmp
3
2
MCM2_2
MCM2_3
MCM2_4
Methanobacteriales
NMD3*
1499
23S rRNA methylase
0293
* Protein affecting ribosome stability and mRNA decay
Methanopyrus
MCM2_2
Archaeoglobus
Methanosarcinales
SpoU rRNA methylase
0565
Mac MCM2_2
Methanospirillum
Thermoplasmatales
Tvo
S13
S4
S11
RpoD
Pto
Halobacteriales
Nph
4
MinD
3
Hma
Hma MCM2_2
Nph
TFB
0642
2
Hsa
O2/light sensing His-kinase

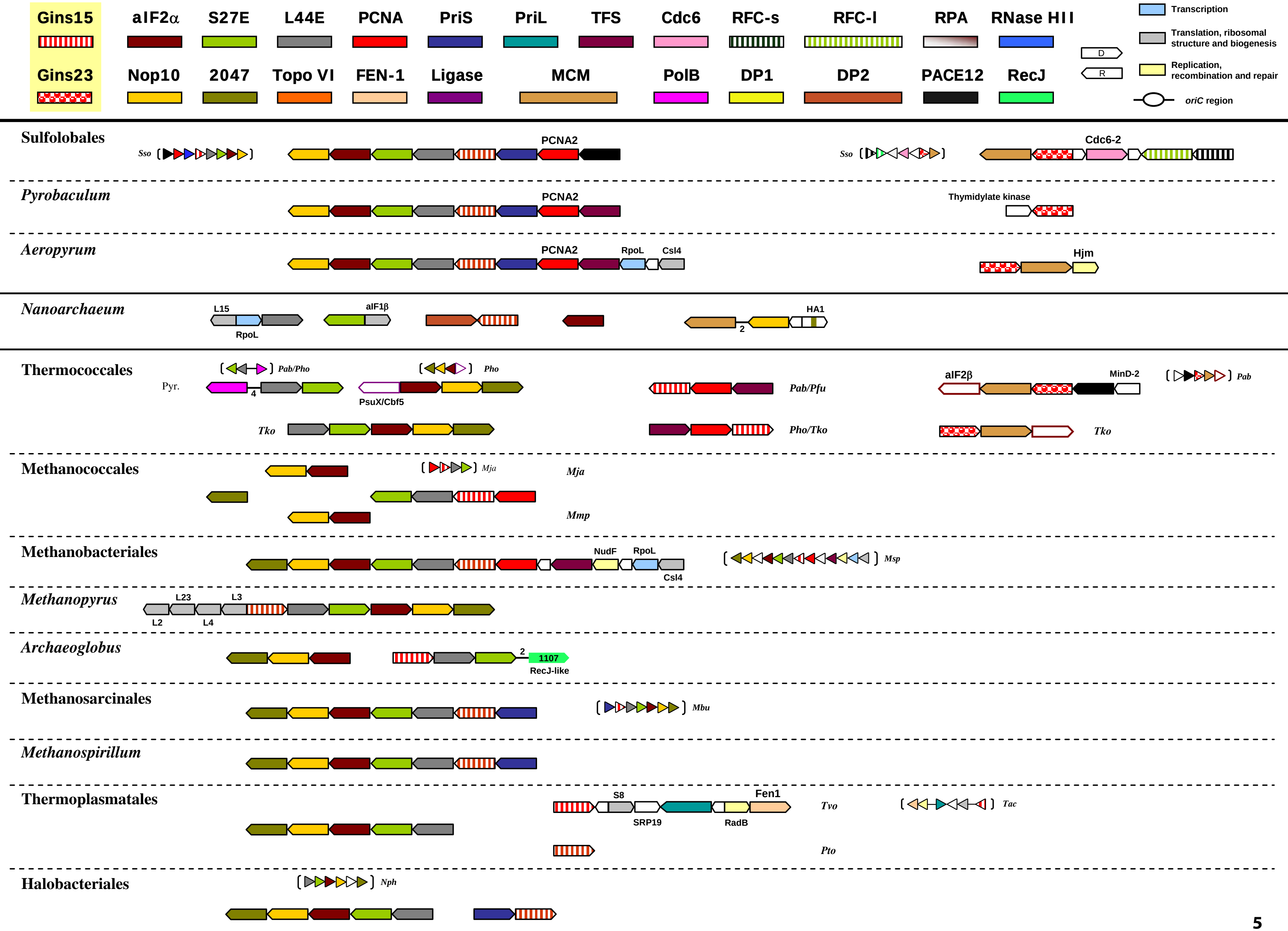

Gins15
aIF2α
S27E
L44E
PCNA
PriS
PriL
TFS
Cdc6
RFC-s
RFC-l
RPA
RNase HII
Gins23
Nop10
2047
Topo VI
FEN-1
Ligase
MCM
PolB
DP1
DP2
PACE12
RecJ
D
R
Transcription
Translation, ribosomal structure and biogenesis
Replication, recombination and repair
oriC region
Sulfolobales
Sso
PCNA2
Cdc6-2
Pyrobaculum
Thymidylate kinase
Aeropyrum
RpoL
Csl4
Hjm
Nanoarchaeum
L15
RpoL
aIF1β
HA1
2
Thermococcales
Pab/Pho
Pho
Pyr.
4
PsuX/Cbf5
Pab/Pfu
aIF2β
MinD-2
Pab
Tko
Pho/Tko
Methanococcales
Mja
Mmp
Methanobacteriales
NudF
Msp
Methanopyrus
L23
L3
L2
L4
Archaeoglobus
1107
RecJ-like
Methanosarcinales
Mbu
Methanospirillum
Thermoplasmatales
S8
Fen1
SRP19
RadB
Tvo
Tac
Pto
Halobacteriales
Nph

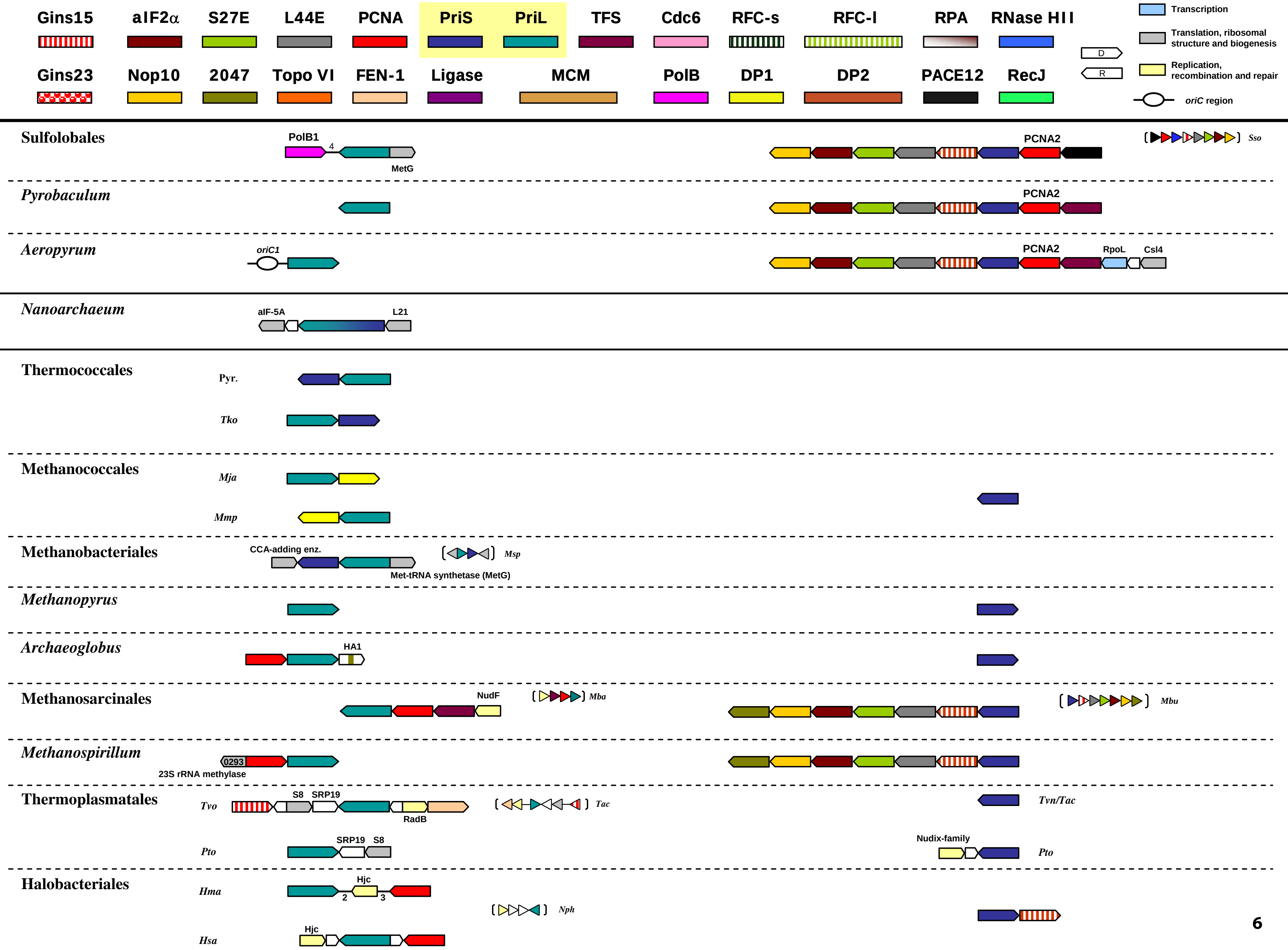

Gins15
aIF2α
S27E
L44E
PCNA
PriS
PriL
TFS
Cdc6
RFC-s
RFC-l
RPA
RNase HII
Gins23
Nop10
2047
Topo VI
FEN-1
Ligase
MCM
PolB
DP1
DP2
PACE12
RecJ
D
R
Transcription
Translation, ribosomal structure and biogenesis
Replication, recombination and repair
oriC region
Sulfolobales
PolB1
4
MetG
PCNA2
Sso
Pyrobaculum
PCNA2
Aeropyrum
oriC1
PCNA2
RpoL
Csl4
Nanoarchaeum
aIF-5A
L21
Thermococcales
Pyr.
Tko
Methanococcales
Mja
Mmp
Methanobacteriales
CCA-adding enz.
Msp
Met-tRNA synthetase (MetG)
Methanopyrus
Archaeoglobus
HA1
Methanosarcinales
NudF
Mba
Mbu
Methanospirillum
0293
23S rRNA methylase
Thermoplasmatales
Tvo
S8
SRP19
RadB
Tac
Tvn/Tac
Pto
SRP19
S8
Nudix-family
Pto
Halobacteriales
Hma
Hjc
2
3
Nph
Hsa
Hjc

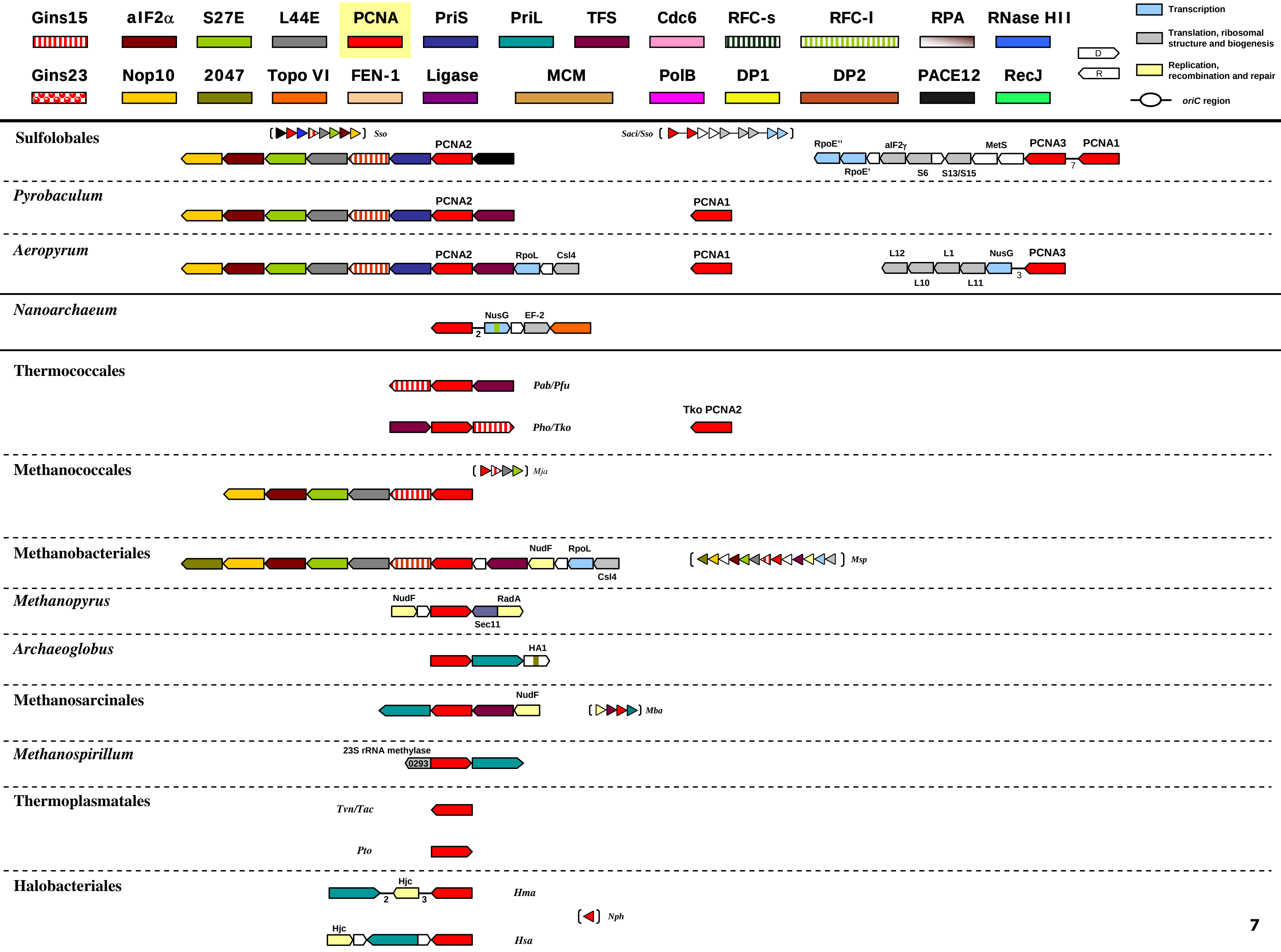
Gins15
aIF2α
S27E
L44E
PCNA
PriS
PriL
TFS
Cdc6
RFC-s
RFC-l
RPA
RNase HII
Gins23
Nop10
2047
Topo VI
FEN-1
Ligase
MCM
PolB
DP1
DP2
PACE12
RecJ
D
R
Transcription
Translation, ribosomal structure and biogenesis
Replication, recombination and repair
oriC region
Sulfolobales
Sso
Saci/Sso
PCNA2
RpoE''
RpoE'
aIF2γ
S6
S13/S15
MetS
PCNA3
PCNA1
7
Pyrobaculum
PCNA2
PCNA1
Aeropyrum
PCNA2
RpoL
Csl4
PCNA1
L12
L10
L1
L11
NusG
PCNA3
3
Nanoarchaeum
NusG
EF-2
2
Thermococcales
Pab/Pfu
Pho/Tko
Tko PCNA2
Methanococcales
Mja
Methanobacteriales
NudF
RpoL
Csl4
Msp
Methanopyrus
NudF
RadA
Sec11
Archaeoglobus
HA1
Methanosarcinales
NudF
Mba
Methanospirillum
23S rRNA methylase
0293
Thermoplasmatales
Tvn/Tac
Pto
Halobacteriales
Hjc
2
3
Hma
Nph
Hjc
Hsa

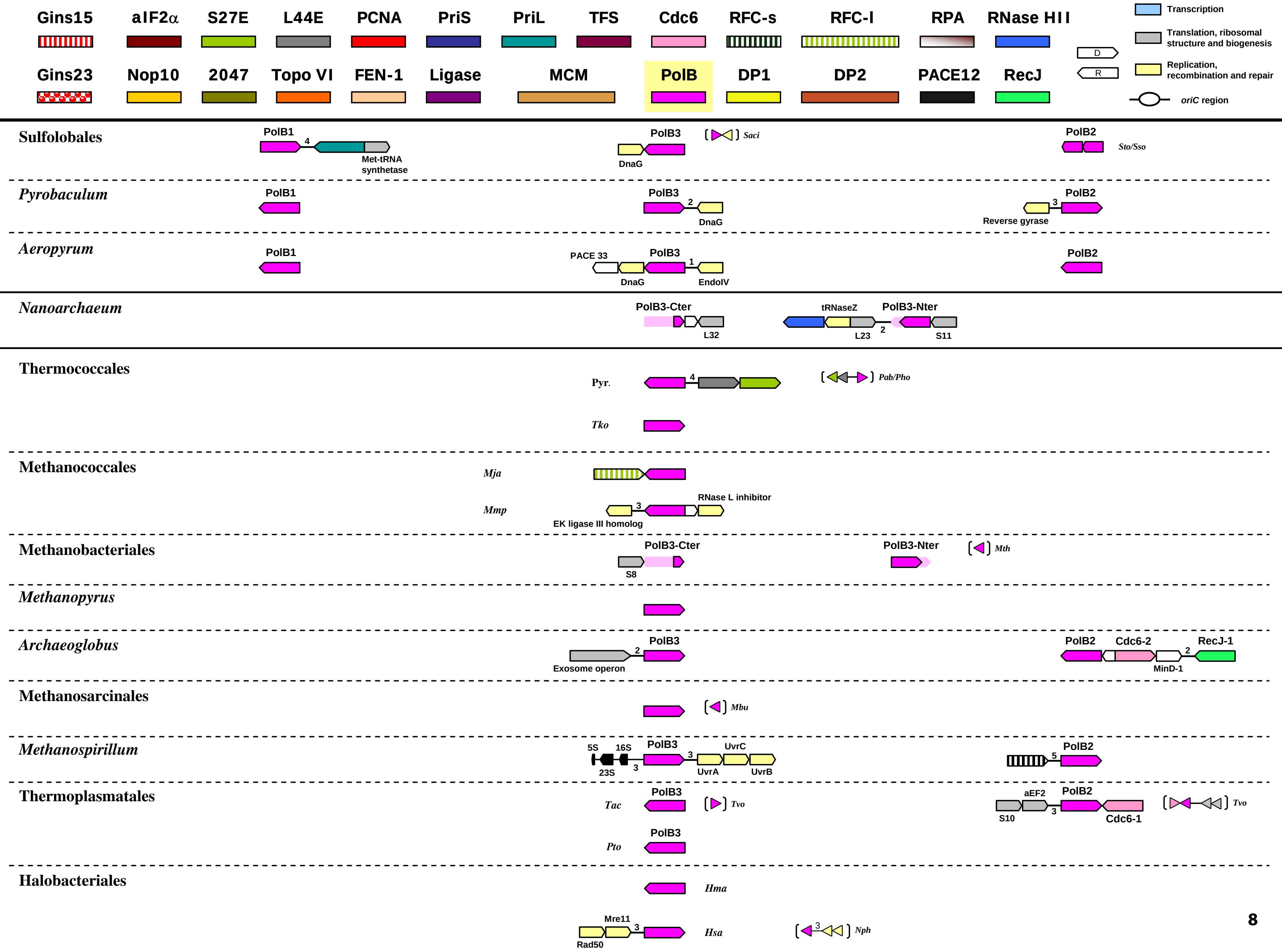
Gins15
aIF2α
S27E
L44E
PCNA
PriS
PriL
TFS
Cdc6
RFC-s
RFC-l
RPA
RNase HII
Gins23
Nop10
2047
Topo VI
FEN-1
Ligase
MCM
PolB
DP1
DP2
PACE12
RecJ
D
R
Transcription
Translation, ribosomal structure and biogenesis
Replication, recombination and repair
oriC region
Sulfolobales
PolB1
Met-tRNA synthetase
PolB3
DnaG
Saci
PolB2
Sto/Sso
Pyrobaculum
Reverse gyrase
Aeropyrum
PACE 33
EndoIV
Nanoarchaeum
PolB3-Cter
L32
tRNaseZ
PolB3-Nter
L23
S11
Thermococcales
Pyr.
Pab/Pho
Tko
Methanococcales
Mja
Mmp
RNase L inhibitor
EK ligase III homolog
Methanobacteriales
S8
Mth
Methanopyrus
Archaeoglobus
Exosome operon
Cdc6-2
RecJ-1
MinD-1
Methanosarcinales
Mbu
Methanospirillum
5S
16S
23S
UvrC
UvrA
UvrB
Thermoplasmatales
Tac
Tvo
aEF2
S10
Cdc6-1
Pto
Halobacteriales
Hma
Mre11
Rad50
Hsa
Nph

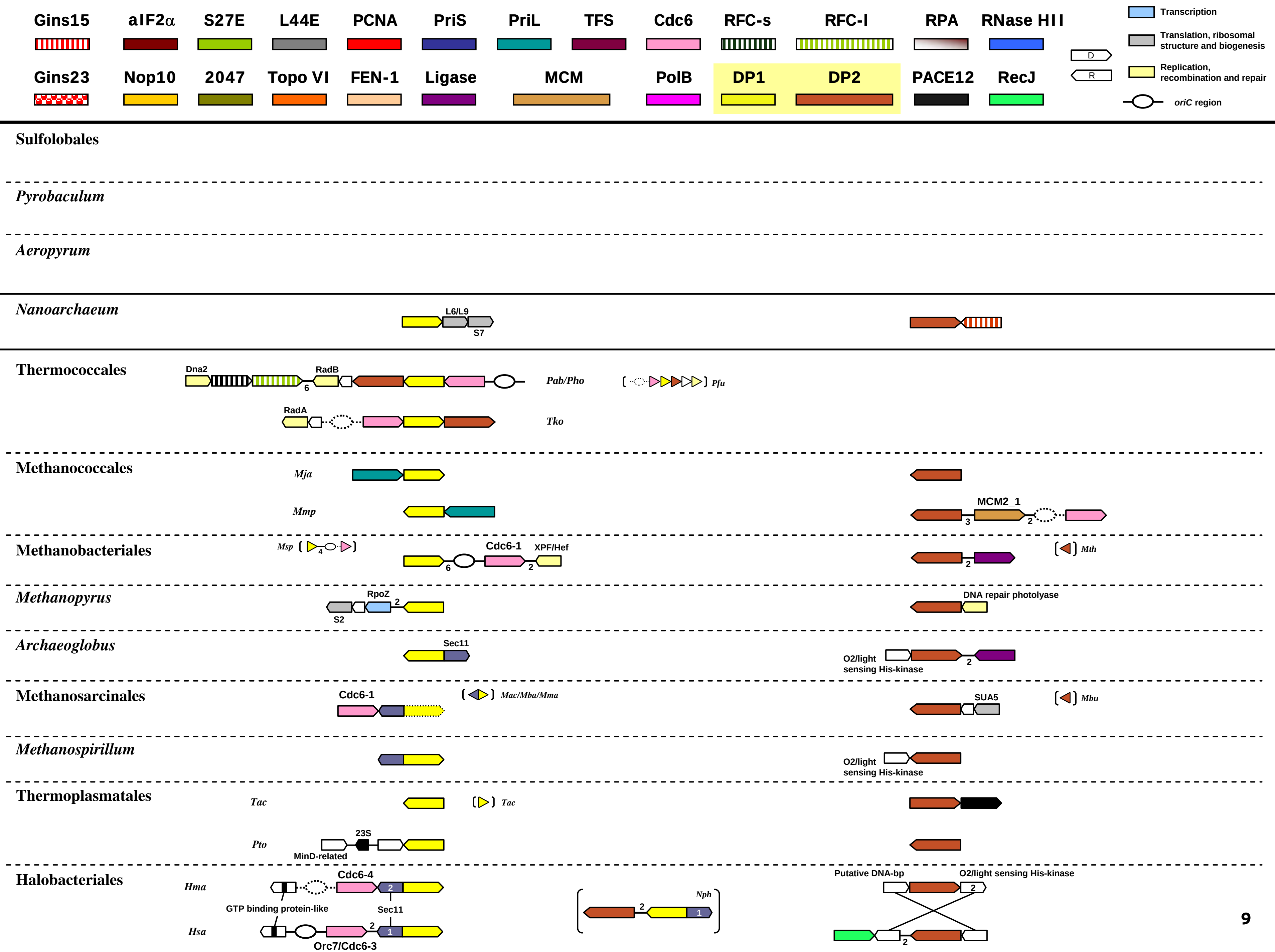
Gins15
aIF2α
S27E
L44E
PCNA
PriS
PriL
TFS
Cdc6
RFC-s
RFC-l
RPA
RNase HII
Gins23
Nop10
2047
Topo VI
FEN-1
Ligase
MCM
PolB
DP1
DP2
PACE12
RecJ
D
R
Transcription
Translation, ribosomal structure and biogenesis
Replication, recombination and repair
oriC region
Sulfolobales
Pyrobaculum
Aeropyrum
Nanoarchaeum
L6/L9
S7
Thermococcales
Dna2
RadB
Pab/Pho
Pfu
RadA
Tko
Methanococcales
Mja
Mmp
MCM2_1
Methanobacteriales
Msp
Cdc6-1
XPF/Hef
Mth
Methanopyrus
RpoZ
S2
DNA repair photolyase
Archaeoglobus
Sec11
O2/light sensing His-kinase
Methanosarcinales
Cdc6-1
Mac/Mba/Mma
SUA5
Mbu
Methanospirillum
O2/light sensing His-kinase
Thermoplasmatales
Tac
Tac
Pto
23S
MinD-related
Halobacteriales
Hma
Cdc6-4
GTP binding protein-like
Sec11
Hsa
Orc7/Cdc6-3
Nph
Putative DNA-bp
O2/light sensing His-kinase

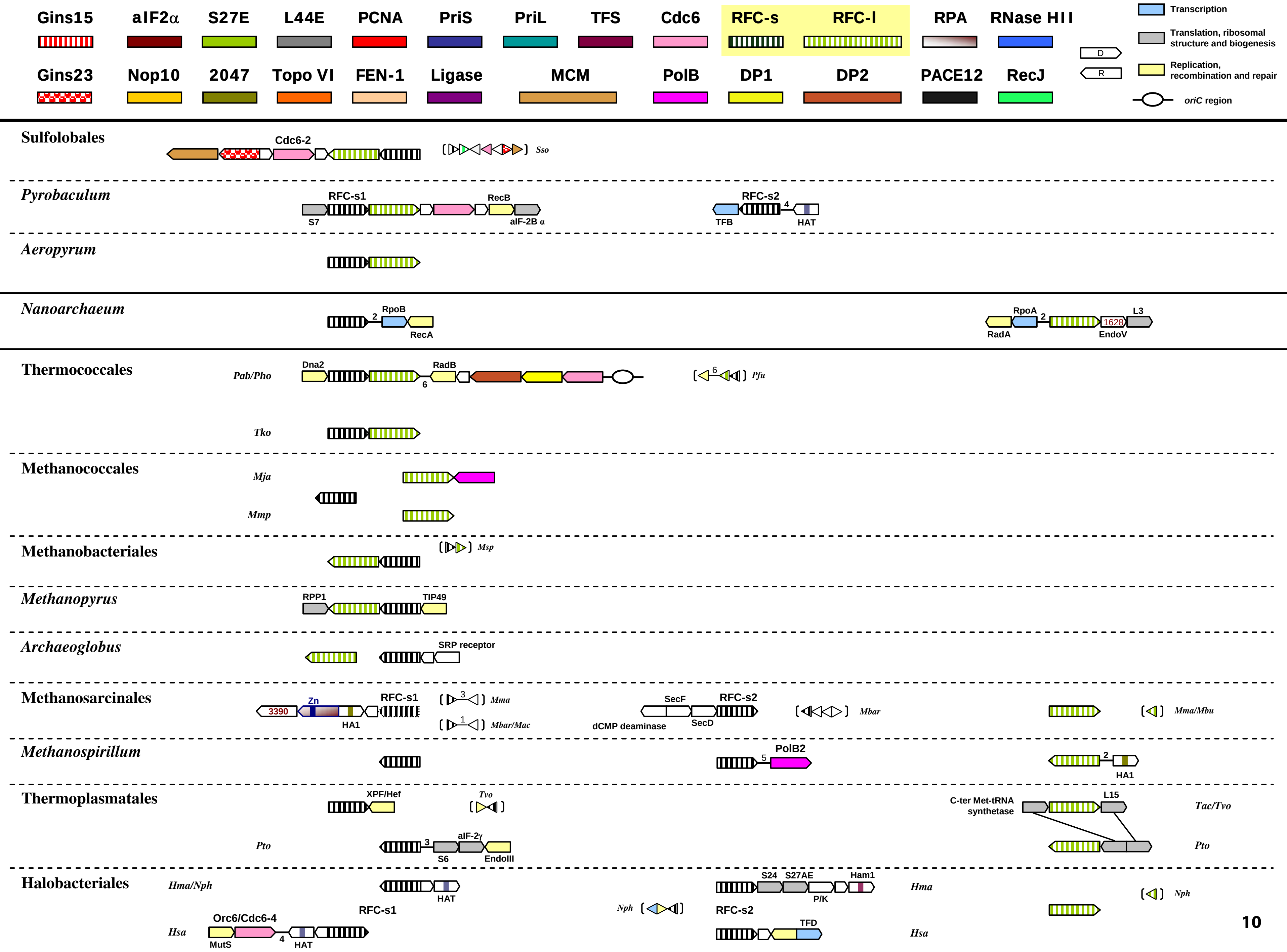
Gins15
aIF2α
S27E
L44E
PCNA
PriS
PriL
TFS
Cdc6
RFC-s
RFC-l
RPA
RNase HII
Gins23
Nop10
2047
Topo VI
FEN-1
Ligase
MCM
PolB
DP1
DP2
PACE12
RecJ
Transcription
Translation, ribosomal structure and biogenesis
Replication, recombination and repair
D
R
oriC region
Sulfolobales
Cdc6-2
Sso
Pyrobaculum
RFC-s1
RecB
S7
aIF-2B α
RFC-s2
TFB
HAT
Aeropyrum
Nanoarchaeum
RpoB
RecA
RpoA
L3
RadA
1628
EndoV
Thermococcales
Pab/Pho
Dna2
RadB
Pfu
Tko
Methanococcales
Mja
Mmp
Methanobacteriales
Msp
Methanopyrus
RPP1
TIP49
Archaeoglobus
SRP receptor
Methanosarcinales
3390
Zn
HA1
RFC-s1
Mma
Mbar/Mac
SecF
RFC-s2
dCMP deaminase
SecD
Mbar
Mma/Mbu
Methanospirillum
PolB2
HA1
Thermoplasmatales
XPF/Hef
Tvo
C-ter Met-tRNA synthetase
L15
Tac/Tvo
Pto
aIF-2γ
S6
EndoIII
Pto
Halobacteriales
Hma/Nph
HAT
S24
S27AE
Ham1
P/K
Hma
Nph
Orc6/Cdc6-4
RFC-s1
Nph
RFC-s2
Hsa
TFD
MutS
HAT
Hsa

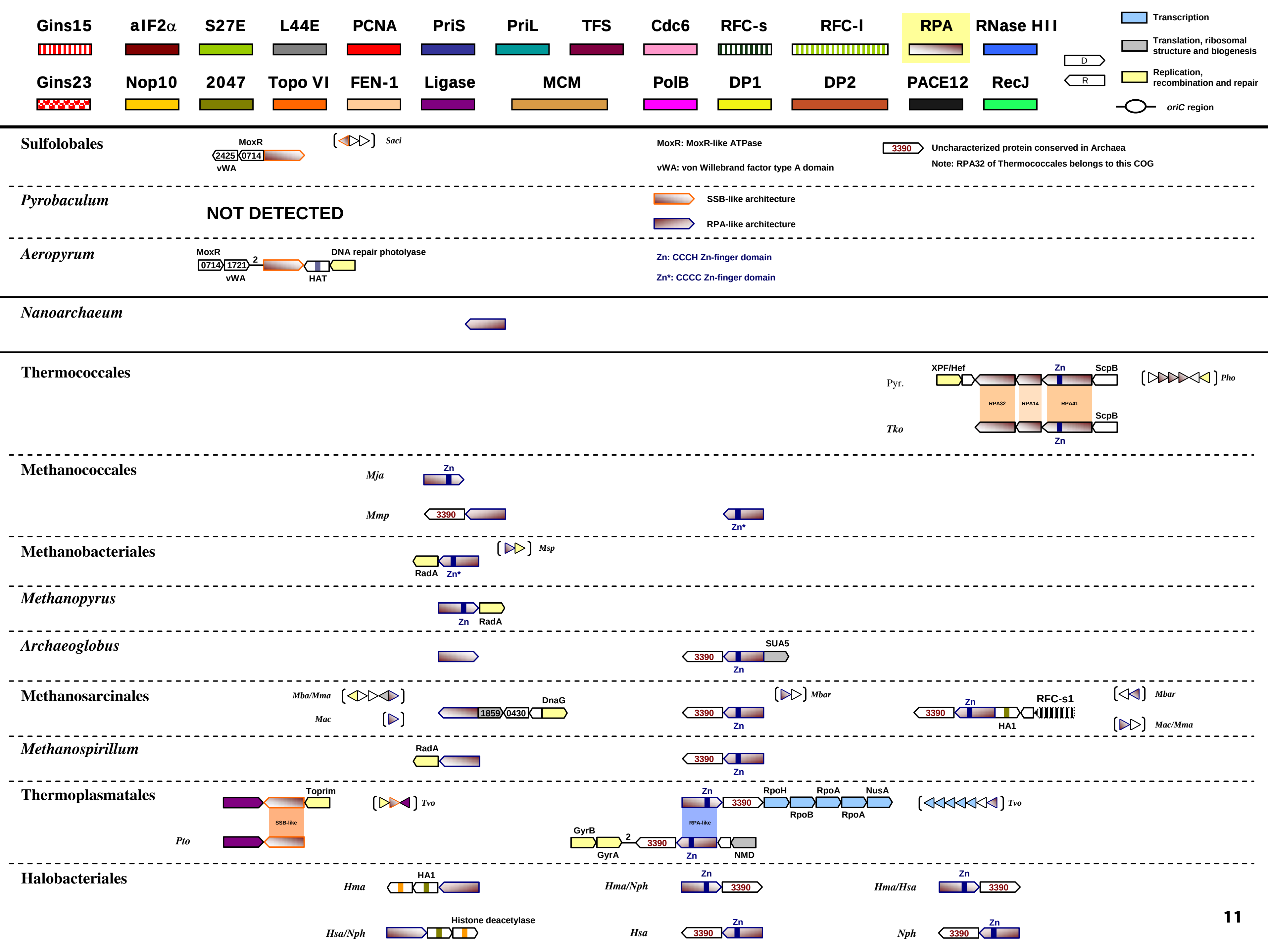

Gins15
aIF2α
S27E
L44E
PCNA
PriS
PriL
TFS
Cdc6
RFC-s
RFC-l
RPA
RNase HII
Gins23
Nop10
2047
Topo VI
FEN-1
Ligase
MCM
PolB
DP1
DP2
PACE12
RecJ
D
R
Transcription
Translation, ribosomal structure and biogenesis
Replication, recombination and repair
oriC region
Sulfolobales
MoxR
2425
0714
vWA
Saci
MoxR: MoxR-like ATPase
vWA: von Willebrand factor type A domain
3390
Uncharacterized protein conserved in Archaea
Note: RPA32 of Thermococcales belongs to this COG
Pyrobaculum
NOT DETECTED
SSB-like architecture
RPA-like architecture
Aeropyrum
MoxR
0714
1721
2
vWA
DNA repair photolyase
HAT
Zn: CCCH Zn-finger domain
Zn*: CCCC Zn-finger domain
Nanoarchaeum
Thermococcales
Pyr.
XPF/Hef
Zn
ScpB
Pho
RPA32
RPA14
RPA41
Tko
ScpB
Zn
Methanococcales
Mja
Zn
Mmp
3390
Zn*
Methanobacteriales
Msp
RadA
Zn*
Methanopyrus
Zn
RadA
Archaeoglobus
SUA5
3390
Zn
Methanosarcinales
Mba/Mma
Mac
1859
0430
DnaG
Mbar
3390
Zn
Zn
RFC-s1
3390
HA1
Mbar
Mac/Mma
Methanospirillum
RadA
3390
Zn
Thermoplasmatales
Toprim
SSB-like
Pto
Tvo
Zn
3390
RpoH
RpoA
NusA
RpoB
RpoA
Tvo
RPA-like
GyrB
2
3390
GyrA
Zn
NMD
Halobacteriales
Hma
HA1
Hma/Nph
Zn
3390
Hma/Hsa
Zn
3390
Hsa/Nph
Histone deacetylase
Hsa
Zn
3390
Nph
Zn
3390

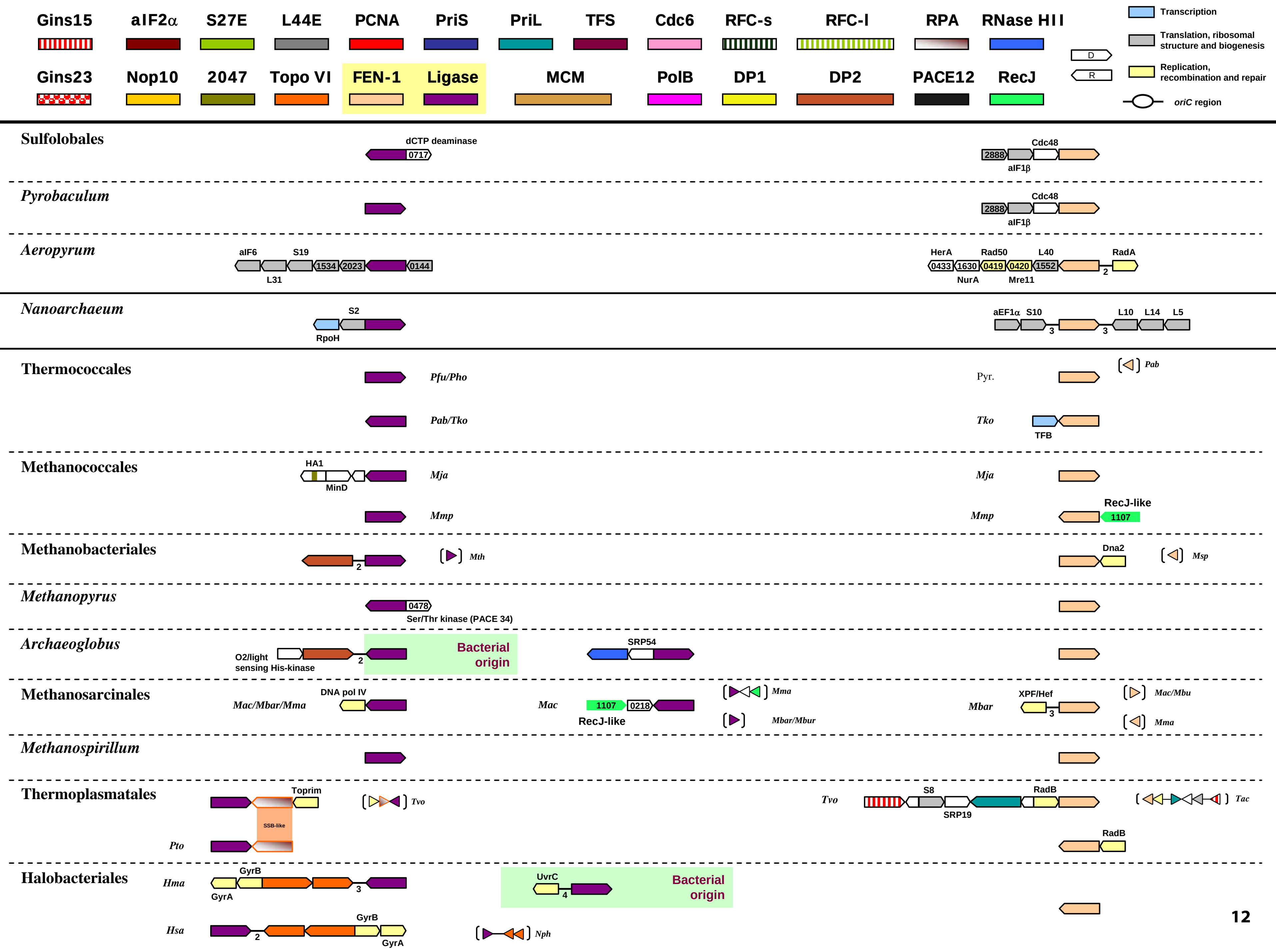

Gins15
aIF2α
S27E
L44E
PCNA
PriS
PriL
TFS
Cdc6
RFC-s
RFC-l
RPA
RNase HII
Gins23
Nop10
2047
Topo VI
FEN-1
Ligase
MCM
PolB
DP1
DP2
PACE12
RecJ
Transcription
Translation, ribosomal structure and biogenesis
Replication, recombination and repair
D
R
oriC region
Sulfolobales
dCTP deaminase
0717
Cdc48
2888
aIF1β
Pyrobaculum
Aeropyrum
aIF6
S19
1534
2023
0144
L31
HerA
Rad50
L40
RadA
0433
1630
0419
0420
1552
NurA
Mre11
Nanoarchaeum
S2
RpoH
aEF1α
S10
L10
L14
L5
Thermococcales
Pfu/Pho
Pyr.
Pab
Pab/Tko
Tko
TFB
Methanococcales
HA1
MinD
Mja
Mmp
RecJ-like
1107
Methanobacteriales
Mth
Dna2
Msp
Methanopyrus
0478
Ser/Thr kinase (PACE 34)
Archaeoglobus
O2/light sensing His-kinase
Bacterial origin
SRP54
Methanosarcinales
DNA pol IV
Mac/Mbar/Mma
Mac
0218
RecJ-like
Mma
Mbar/Mbur
Mbar
XPF/Hef
Mac/Mbu
Methanospirillum
Thermoplasmatales
Toprim
SSB-like
Tvo
Pto
S8
SRP19
RadB
Tac
Halobacteriales
Hma
GyrB
GyrA
UvrC
Bacterial origin
Hsa
Nph

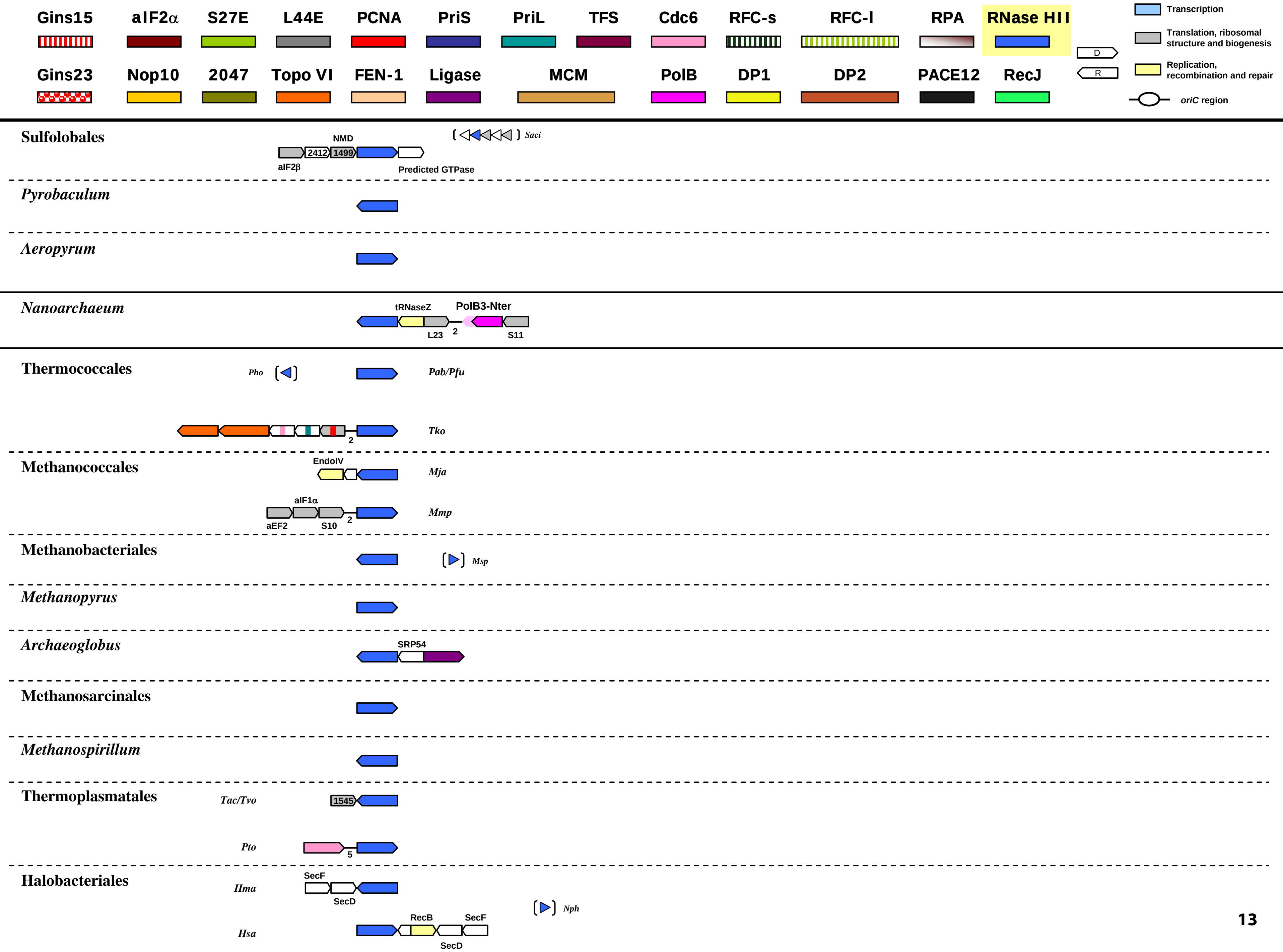
Gins15
aIF2α
S27E
L44E
PCNA
PriS
PriL
TFS
Cdc6
RFC-s
RFC-l
RPA
RNase HII
Gins23
Nop10
2047
Topo VI
FEN-1
Ligase
MCM
PolB
DP1
DP2
PACE12
RecJ
D
R
Transcription
Translation, ribosomal structure and biogenesis
Replication, recombination and repair
oriC region
Sulfolobales
NMD
2412
1499
aIF2β
Predicted GTPase
Saci
Pyrobaculum
Aeropyrum
Nanoarchaeum
tRNaseZ
PolB3-Nter
L23
2
S11
Thermococcales
Pho
Pab/Pfu
2
Tko
Methanococcales
EndoIV
Mja
aIF1α
aEF2
S10
2
Mmp
Methanobacteriales
Msp
Methanopyrus
Archaeoglobus
SRP54
Methanosarcinales
Methanospirillum
Thermoplasmatales
Tac/Tvo
1545
Pto
5
Halobacteriales
SecF
Hma
SecD
Nph
RecB
SecF
Hsa
SecD

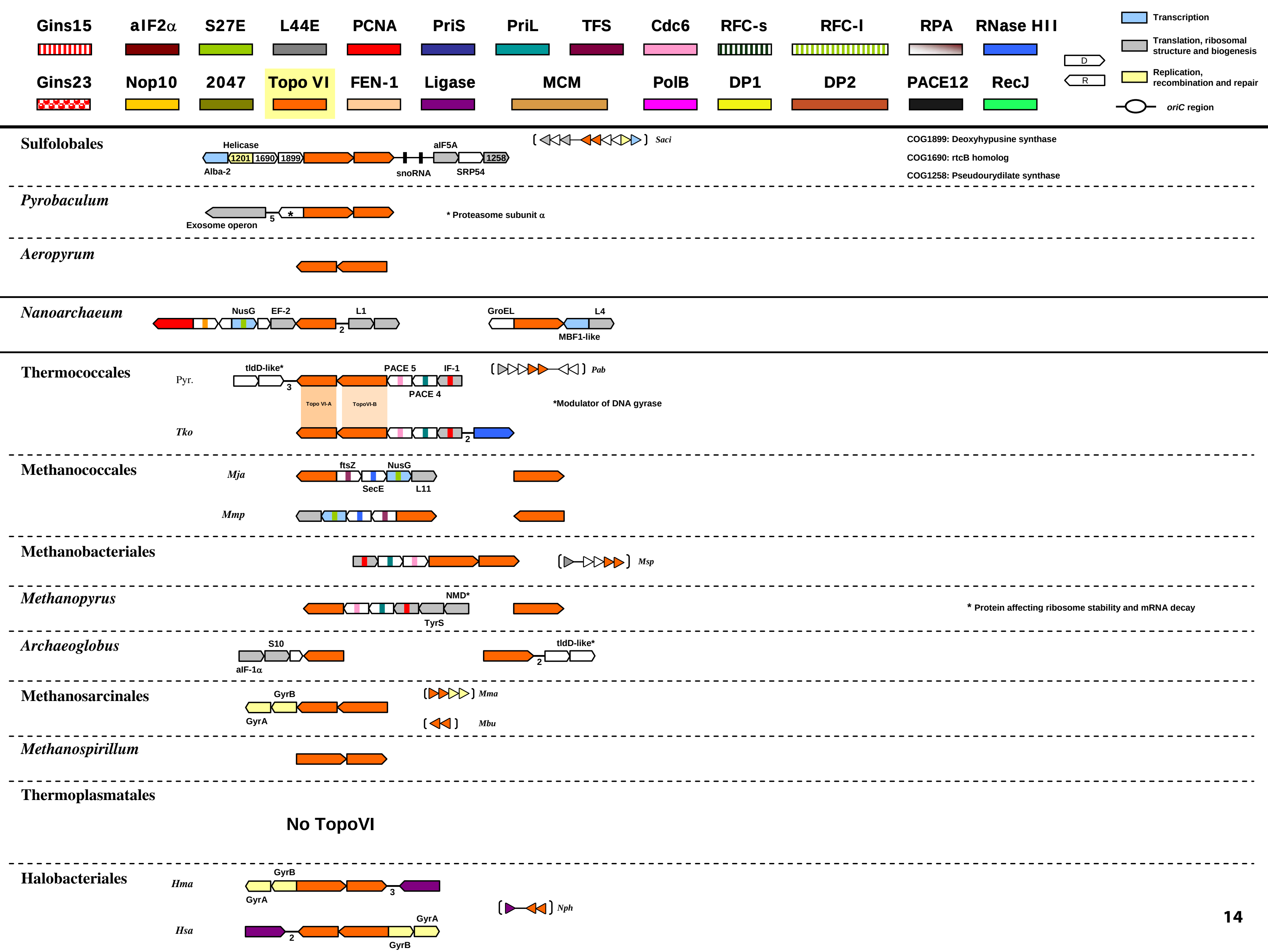
Gins15
aIF2α
S27E
L44E
PCNA
PriS
PriL
TFS
Cdc6
RFC-s
RFC-l
RPA
RNase HII
Gins23
Nop10
2047
Topo VI
FEN-1
Ligase
MCM
PolB
DP1
DP2
PACE12
RecJ
D
R
Transcription
Translation, ribosomal structure and biogenesis
Replication, recombination and repair
oriC region
Sulfolobales
Helicase
1201
1690
1899
aIF5A
1258
Alba-2
snoRNA
SRP54
Saci
COG1899: Deoxyhypusine synthase
COG1690: rtcB homolog
COG1258: Pseudourydilate synthase
Pyrobaculum
5
*
Exosome operon
* Proteasome subunit α
Aeropyrum
Nanoarchaeum
NusG
EF-2
L1
2
GroEL
L4
MBF1-like
Thermococcales
Pyr.
tldD-like*
3
PACE 5
IF-1
PACE 4
Pab
Topo VI-A
TopoVI-B
*Modulator of DNA gyrase
Tko
2
Methanococcales
Mja
ftsZ
NusG
SecE
L11
Mmp
Methanobacteriales
Msp
Methanopyrus
NMD*
TyrS
* Protein affecting ribosome stability and mRNA decay
Archaeoglobus
S10
aIF-1α
tldD-like*
2
Methanosarcinales
GyrB
GyrA
Mma
Mbu
Methanospirillum
Thermoplasmatales
No TopoVI
Halobacteriales
Hma
GyrB
GyrA
3
Nph
Hsa
2
GyrA
GyrB
