# Supplementary material for: Genomic context analysis in Archaea suggests previously unrecognized links between DNA replication and translation
Source: Genome Biol. 2008 Apr 9;9(4):R71. doi: 10.1186/gb-2008-9-4-r71 (PMC2643942; doi:10.1186/gb-2008-9-4-r71)
Supplement: Additional data file 3 — Description of and discussion about genomic associations of DNA replication genes with genes coding for archaeal homologs of DNA repair/recombination proteins. [file gb-2008-9-4-r71-S3.doc]

**Additional data file 3**

In addition to the conserved associations described in the section Results and Discussion, we observed that DNA replication genes frequently lie together or are often adjacent to genes encoding proteins involved in other informational processes (i.e., DNA repair, DNA recombination, translation). These clusters are usually small (two genes) and generally not conserved form one genome to another (except for those of closely related species). However, these associations make *a priori* sense in term of functionality. Furthermore, it might be significant that a gene coding for a given protein (for instance a repair protein) co-localizes in different archaea with various DNA replication genes, suggesting a possible loose network of interactions between the gene products. These clusters are therefore described briefly as possible guides for future experimental studies (see additional data file 2 for illustrations).

***Genomic context connecting genes coding for DNA replication proteins possibly involved in the processing of Okazaki’s fragments***

Proteins known to be involved in the processing of Okazaki fragments in Eukarya (RNase HII, FEN-1, DNA ligase, Dna2) have orthologues in archaea. True homologues of eukaryotic Dna2 are only present in Halobacteriales. However, other archaea encode a protein formed by the fusion of two domains related to the eukaryotic Dna2: the first one is homologous to the helicase domain; the second one is a highly divergent form of the RecB-like domain. Genomic context analysis revealed a weak network linking RNase HII, Dna2, FEN-1, and DNA ligase to one another and to other DNA replication proteins. For instance, the gene encoding the archaeal Dna2-like protein is located close to the gene encoding FEN-1 in Methanobacteriales, the gene for Cdc6 in *Picrophilus torridus* and is located in the vicinity of the *Pyrococcus* replication islands containing *oriC.* The gene encoding FEN-1 is located a few genes away from the genes encoding PriL and Gins15 in *Thermoplasma volcanium*. The DNA ligase gene co-localizes with the gene encoding the single-stranded binding protein in Thermoplasmatales, the gene for DP2 in *Methanosphaera stadtmanae*, the gene coding for PriL in *Archaeoglobus fulgidus*, and the gene encoding the RNase HII in *Archaeoglobus fulgidus*. Finally, the gene coding for the RNase HII co-localizes with the gene encoding PolB in *Nanoarchaeum equitans*.

***Clusters of genes encoding proteins that could be involved in DNA replication and in DNA recombination/repair***

We have detected several clusters of genes encoding DNA replication and repair proteins. Although they are usually not conserved between distantly related lineages, these gene associations make sense in term of functionality. Several DNA replication genes are located in the vicinity of genes encoding DNA glycosylases specific for damaged bases (homologues of *E. coli* endonuclease III and endonuclease V) or apurinic sites (homologues of *E. coli* endonuclease IV) suggesting that these DNA replication proteins could work in base excision repair (BER).The gene encoding an Endo V homologue is close to the gene encoding RFC-l in *Nanoarchaeum equitans*, whereas the gene encoding Endo IV homologue is close to the gene encoding PolB in *Aeropyrum pernix*, the gene coding for RNase HII in *Methanocaldococcus jannashii* and the gene for RecJ in *Methanococcus maripaludis.* Finally, a gene encoding an Endo III homologue is close to the gene encoding Cdc6 in *Aeropyrum pernix.* The gene encoding the archaeal DNA ligase co-localizes in Methanosarcinales with the gene encoding a DNA polymerase of the X family of probable bacterial origin. These DNA polymerases, whose prototypes are *E. coli* DNA polymerase IV and eukaryotic DNA polymerase beta, are used in connection with DNA ligase in nucleotide excision repair (NER) and/or in repair of double-stranded breaks by Non Homologous End Joining (NHEJ) (for a review, see [80]) Other associations of an archaeal gene with a gene of bacterial origin are suggestive of NER. Hence, the gene encoding PolB is located close to the genes encoding the NER proteins UvrABC (also of bacterial origin) in *Methanospirillum hungatei*, whereas the gene encoding PolD is close to a gene encoding a DNA ligase of bacterial origin in *Archaeoglobus fulgidus* and of a gene encoding an UvrC-like protein (also of bacterial origin) in *Haloarcula marismortui*. It should be interesting to know if Archaea have recruited bacterial DNA repair genes introduced by gene transfer to produce new repair pathways by combining their protein products with *bona fide* archaeal proteins or if these bacterial proteins have displaced the original archaeal protein in repair pathways that previously existed.

We also notice associations of DNA replication genes with genes encoding proteins that could be involved in the repair of stalled or broken replication forks by homologous recombination. This mechanism can be divided into two steps: processing of the stalled or broken replication fork to produce a suitable substrate for the recombination enzymes, followed by classical homologous recombination involving the formation and the resolution of Holliday junctions. Two archaeal proteins that have been characterized biochemically in *Pyrococcus furiosus* could be involved in the processing of stalled replication fork: the nuclease/helicase Hef and the helicase Hel308a/Hjm (a RecQ analogue) [81-83]. The nuclease domain of Hef is homologous to the nuclease domain of the eukaryotic XPF protein, which is involved in nucleotide excision repair. The Hef/XPF protein itself is only found in euryarchaea, whereas the crenarchaea only contain a homologue of the XPF domain (reviewed in [29]). The possible participation of Hel308a/Hjm and Hef/XPF proteins in the repair of stalled replication fork is supported by the fact that PCNA stimulates *in vitro* the helicase function of Hel308a/Hjm in *P. furiosus* [82] and by the observation that *S. solfataricus* XPF activity is dependent on PCNA and modulated by the single-stranded binding protein found in this organism [84]. Our genome context analysis also suggests a loose connection of these two proteins to the DNA replication factory since the genes encoding Hef/XPF and Hel308a/Hjm sometimes co-localize with genes encoding DNA replication proteins. The gene encoding Hel308a/Hjm is adjacent to the gene encoding MCM in *A. pernix* and *P. aerophilum*, whereas the gene encoding Hef/XPF co-localizes with the genes encoding RFC-s in *Thermoplasma* species, FEN-1 in *Methanosarcina barkeri*, and RPA in the three *Pyrococcus*. The latter gene association indicates that the modulation of activity of Hef/XPF proteins by single-stranded binding proteins may be a common regulatory feature of this repair enzyme.

Another protein which could participate in the repair of stalled replication fork is RecJ (see Results and discussion section). Interestingly, the gene encoding RecJ is located close to the *mre11* operon in Methanosarcinales and Thermoplasmatales (not shown). This archaeal operon is formed by the association of four genes encoding Mre11, Rad50, the nuclease HerA and the bipolar helicase HerA. It has been shown that these genes are co-transcribed in *Sulfolobus* [19]. The association of RecJ with the *mre11* operon is probably significant since in eukaryotes, Rad50 and Mre11 are involved in the processing of double-stranded break that produce protruding single-stranded 3’ tails to initiate homologous recombination (a role analogous to the role of RecBCD in Bacteria). In eukaryotes, the properties of the Rad50 and Mre11 proteins cannot account for the formation of these tails. In contrast, this can be easily explained (at least theoretically) in archaea by the combined activities of HerA and NurA [18] [19]. In addition to its association with the gene encoding RecJ, we found several genomic contexts supporting the idea that the *mre11* operon is involved in DNA repair. The complete *mre11* operon is close to the genes encoding FEN-1 and RadA in *Aeropyrum pernix,* whereas the genes encoding Mre11 and Rad50 are close to the gene encoding PolB3 in Halobacteriales. All these observations suggest that RecJ and the proteins of the *mre11* operon could be involved in the processing of stalled replication forks to provide suitable substrates for the recombinases. Archaeal genomes harbor one orthologue of the universal recombinase RecA/Rad51, called RadA, and an archaeal-specific paralogue of RadA, called RadB. Several genomic contexts suggest that these two proteins could be associated to the replication fork. Indeed, the gene encoding RadA shares the same locus with the genes coding for FEN-1 in *Aeropyrum pernix*, RFC-l in *Nanoarchaeum equitans*, PCNA in *Methanopyrus kandleri*, RPA in Methanobacteriales, *Methanospirillum hungatei* and *Methanopyrus kandleri*. The gene encoding RadB is present in the DNA replication islands of the three *Pyrococcus* species and is adjacent to the genes encoding PriL and FEN-1 in *Thermoplasma* species. The relevance of the genomic co-localization of the genes encoding RadB and DP1 is supported by two-hybrid analysis in yeast suggesting that these two proteins interact in *Pyrococcus furiosus* [85].

The last step of homologous recombination is the resolution of the Holliday junction. Archaea encode an archaeal-specific Holliday junction resolvase Hjc (Holliday junction cleavage) that has been discovered by a biochemical approach [86]. The gene encoding Hjc is close to the genes encoding PCNA and PriL in *Halobacterium salinarum* and *Haloarcula marismortui*. This genomic association, observed in two related euryarchaeotes, is interesting since it has been recently shown that PCNA activates Hjc *in vitro* in the crenarchaeote *Sulfolobus solfataricus* [87]. It is tempting to suggest that all the gene associations described above (although never conserved) reflect the existence of functional connection between DNA replication factors and all the proteins involved in the repair of stalled replication fork, from the enzymes involved in the processing of the broken fork to those involved in the final recombination step.

***Genomic associations of DNA replication genes and genes encoding proteins involved in translation***

We frequently observed genes encoding various ribosomal proteins in the vicinity of DNA replication genes (often as immediate neighbors). However, besides the association with L44E and S27E described in the Results and Discussion section, these clusters are not conserved in distantly related genomes and involve different ribosomal proteins encoding genes. One exception concerns the gene encoding the ribosomal protein S10E which is located close to the genes encoding PolB and Cdc6 in *T. volcanium*, adjacent to a gene encoding the A subunit of Topo VI in *Archaeoglobus* and in the vicinity of the gene for FEN-1 in *N. equitans*. Finally, we have noticed that the gene encoding the archaeal RecJ protein frequently associates with the genes encoding the ribosomal proteins S15P and S3AE.

Curiously, in euryarchaeal genomes DNA replication/repair genes are also often adjacent to genes encoding components of the apparatus involved in the synthesis and localization of membrane proteins. Hence, the gene encoding the signal recognition particle SRP54 is adjacent to the gene encoding DNA ligase and RNase HII in *Archaeoglobus fulgidus* and TFS in *Picrophilus torridus*. The gene encoding the protein SRP19 neighbors the gene encoding PriL in *Thermoplasma volcanium*. The gene encoding the SRP receptor co-localizes with the genes encoding the two RFC subunits in *Archaeoglobus fulgidus*. Finally, the gene encoding the signal peptidase involved in the maturation of membrane proteins (*sec11*) co-localizes with the cluster of genes encoding PCNA, RadA, and NudF in *Methanopyrus kandleri*, with the gene encoding DP1 in five euryarchaeal lineages and with the gene for DP2 in *Natronomonas pharaonis*; in Methanosarcinales and in two Halobacteriales, the genes encoding Sec11 and DP1 are close to the gene encoding Cdc6.
