# Supplementary material for: Genomic context analysis in Archaea suggests previously unrecognized links between DNA replication and translation
Source: Genome Biol. 2008 Apr 9;9(4):R71. doi: 10.1186/gb-2008-9-4-r71 (PMC2643942; doi:10.1186/gb-2008-9-4-r71)

Additional data file 4

Statistical significance of 32 representative gene clusters.

(a) Clusters and prevalence indexes for 32 gene clusters. The table shows 32 gene clusters analyzed in this work; their presences and absences in each of the 27 archaeal genomes; their sizes; their prevalence indexes; and the percentage of the frequency distributions where they are found (frequency scores). Gene clusters with a frequency score of 5% or less are statistically supported (indicated by an asterisk). The distributions were calculated for each size of gene clusters using the data from the 10,000 simulations as explained in the Materials and methods section (results of these simulations are shown in Figure 5). (b) Examples of frequency distributions for clusters of two (left) and three or more genes (right). The red areas represent the 5% and less of the right part of the distributions: the statistically significant area.

(a)

Gene clusters	Presence in genomes	Absence in genomes	Index	# genes in cluster	Frequency score
2047 + Nop10	19	8	11	2	11%
Nop10 + aIF2a	26	1	25	2	1% *
aIF2a + S27E	21	6	15	2	14%
S27E + L44E	26	1	25	2	1% *
L44E + Gins15	16	11	5	2	17%
Gins15 + PriS	13	14	-1	2	30%
Gins15 + PCNA	8	19	-11	2	55%
PriS + PCNA	5	22	-17	2	75%
PCNA + TFS	10	17	-7	2	57%
TFS + NudF	6	21	-15	2	70%
PriL + PCNA	6	21	-15	2	70%
aIF2b + MCM	6	21	-15	2	31%
MCM + Gins23	8	19	-11	2	55%
Gins15 + PriS + PCNA	5	22	-17	3	9%
Gins15 + PriS + PCNA + TFS	2	25	-23	3	26%
Gins15 + PCNA + TFS	4	23	-19	3	88%
PCNA + TFS + NudF	4	23	-19	3	88%
Gins15 + PriS + PCNA + TFS	2	25	-23	4	25%
PriL + PCNA + TFS + NudF	4	23	-19	4	6%
2047 + Nop10 + aIF2a	19	8	11	3	3% *
2047 + Nop10 + aIF2a + S27E	15	12	3	4	2% *
2047 + Nop10 + aIF2a + S27E + L44E	15	12	3	5	2% *
2047 + Nop10 + aIF2a + S27E + L44E + Gins15	8	19	-11	6	2% *
2047 + Nop10 + aIF2a + S27E + L44E + Gins15 + PriS	5	22	-17	7	2% *
2047 + Nop10 + aIF2a + S27E + L44E + Gins15 + PCNA	2	25	-23	7	12%
Nop10 + aIF2a + S27E	21	6	15	3	2% *
Nop10 + aIF2a + S27E + L44E	21	6	15	4	2% *
Nop10 + aIF2a + S27E + L44E + Gins15	14	13	1	5	3% *
Nop10 + aIF2a + S27E + L44E + Gins15 + PriS	10	17	-7	6	2% *
Nop10 + aIF2a + S27E + L44E + Gins15 + PCNA	3	24	-21	6	5% *
Nop10 + aIF2a + S27E + L44E + Gins15 + PriS + PCNA	5	22	-17	7	2% *
Nop10 + aIF2a + S27E + L44E + Gins15 + PriS + PCNA + TFS	2	25	-23	8	10%

(b)

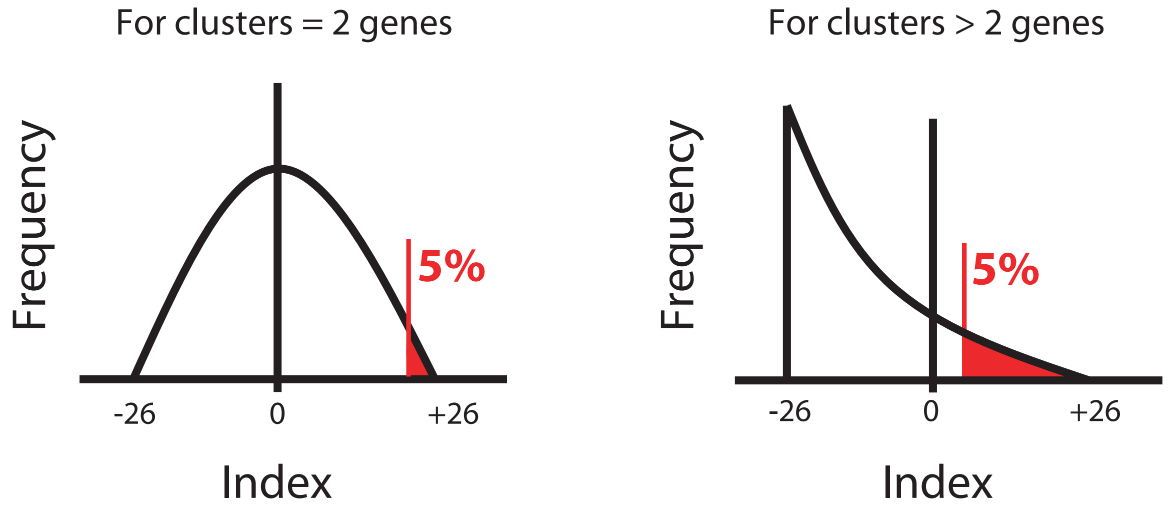

Supplement: Additional data file 4 — Prevalence indexes of gene clusters and frequency distributions of clusters of two genes and clusters of more than two genes. [file gb-2008-9-4-r71-S4.pdf]
